# The effect of soaking heat-polymerized acrylic resin denture base in avocado seed extract ( *Persea americana *Mill.) on the inhibition of denture-plaque microorganisms biofilm growth

**DOI:** 10.12688/f1000research.152800.2

**Published:** 2024-10-29

**Authors:** Thalia Angela, Siti Wahyuni, Susanna Halim

**Affiliations:** 1 Dental Undergraduate Study Program, Faculty of Dentistry, University of Sumatera Utara, Medan, North Sumatra, Indonesia; 2 Department of Prosthodontics, University of Sumatera Utara, Medan, North Sumatra, Indonesia; 3 Faculty of Medicine, Dentistry and Health Sciences, Prima Indonesia University, Medan, North Sumatra, Indonesia

**Keywords:** heat polymerized acrylic resin, avocado seed extract, mono-species biofilm, polymicrobial biofilm, denture plaque, MBIC

## Abstract

**Background:**

Heat polymerized acrylic (HPA) resins are known to have high porosity that contributes to increased surface roughness and microcrack formation in stress areas. This facilitates the attachment and growth of polymicrobial biofilms contributing to increased antimicrobial resistance. Many research had been carried out on avocado seeds, but no research that studies avocado seeds effect on denture-plaque microorganism biofilm on HPA resin has been found.

**Methods:**

This study used 144 samples (n=144), namely HPA resin discs covered with mono-species and polymicrobial biofilms. The discs were soaked for 8 hours in the 5%, 10%, 15%, 20% avocado seed extract, positive control (alkaline peroxide), and negative control (aquadest). Each disc was shaken with a vortex mixer for 1 minute, and 100 μL was added into 96-well microplates with three times repetition and incubated for 24 hours. A microtiter plate biofilm formation assay was then conducted The inhibition values were determined from the percentage inhibition value formula which required absorption values from a microplate reader (595 nm). The research data were analyzed using a univariant test, and a one-way ANOVA test, accompanied by Welch ANOVA on non-homogenous data.

**Results:**

In this research, it was found that the MBIC _50_ of avocado seed extract against the mono-species of *C. albicans* (5%), *C. glabrata* (5%), *A. odontolyticus* (15%), *S. gordonii* (15%), *S. aureus* (10%), while against polymicrobial was 20%. There was a significant effect of soaking HPA resin in avocado seed extract on the inhibition of mono-species and polymicrobial biofilms with a value of p<0.001 (p<0.05).

**Conclusion:**

The MBIC _50_ of avocado seed extract in polymicrobial biofilm group was higher than that in the mono-species biofilm groups. Although alkaline peroxide showed higher inhibition value than that of the MBIC _50_ in polymicrobial biofilm group, 20% avocado seed extract was concluded effective as it inhibited >50% polymicrobial biofilm.

## Introduction

The denture base is a part of the denture which rests on the supporting tissue and serves as a place for the arrangement of tooth elements. ^1^ Denture base materials vary greatly, but the most commonly used and popular material is polymethyl methacrylate acrylic resin (PMMA) with more than 95% of fabricated denture bases are made from acrylic resin. ^2^^,^^3^ Acrylic resin itself has various types, one of which is heat polymerized acrylic resin. ^4^ Heat polymerized acrylic (HPA) resin has better strength properties and a higher degree of polymerization, less residual monomer, and more stable color. ^5^^,^^6^ However, it still has limitations, some of which have porous properties and high surface roughness which can increase the attachment of fungal and bacterial biofilms. ^4^

Colonization in a biofilm requires strong attachment of oral microorganisms by integrating into the salivary pellicle to form plaque on the denture material. Surface roughness and surface free energy are two factors that can promote plaque development. ^7^ Surface roughness of acrylic resin can be reduced by adequate polishing. However, this cannot prevent the build-up of plaque on the denture due to the presence of microporosity in the acrylic resin which cannot be completely avoided. ^4^ This area of porosity becomes an environment which can protect microorganisms in the biofilm. ^7^ In addition, the abiotic surface of the denture causes less exposure of the denture biofilm to the host immune system so that microorganisms can grow without hindrance and have sufficient time to develop into plaque with varying compositions. ^8^

O’Donnell et al. (2015) stated that the composition and diversity of dental plaque was different from denture plaque. Denture plaque in the oral cavity was found to be colonized by *Candida* spp. against the denture surface which co-aggregated with bacteria in the oral cavity. ^8^ As many as 60% to 100% of denture wearers were found to carry *Candida* in the oral cavity in higher quantities compared to those who did not wear dentures. ^8^^,^^9^ The commonly found *Candida* species in denture plaque is *Candida albicans.* Another *Candida* species that is found in denture plaque and increases with age is *Candida glabrata.* Together with *C. albicans*, these two fungal species can form more pathogenic and invasive biofilms and increase the severity of denture stomatitis. ^8^ Several studies have found that denture plaque compared to dental plaque has a higher proportion of obligate anaerobic *Actinomyces* spp., a low proportion of Gram-negative rods, and the common presence of *Staphylococcus aureus*. ^10^ Shi et al. (2016) found that the genus of bacteria which was most commonly found on both surfaces of denture teeth and remaining natural teeth was the genus *Actinomyces*, followed by *Streptococcus*, *Veillonella*, *Capnocytophaga*, *Neisseria*, *Prevotella*, and *Corynebacterium.* ^11^ Based on the genus mentioned, the bacterial species which will be used in this study were *Actinomyces odontolyticus*, *Staphylococcus aureus*, and *Streptococcus gordonii.*

The presence of these three bacteria in dentures can increase the virulence of *C. albicans* thereby increasing damage and invasion of mucosal tissue which increases the risk of denture stomatitis. Morse et al. (2018) found a significant increase in tissue damage from mixed *Candida* and bacterial biofilms where the composition of the biofilm was broadly the composition of denture plaque. ^8^^,^^12^ The difference between biofilms and planktonic bacteria or fungi is that biofilms are a community of microbial cells enveloped in a matrix, while planktonic bacteria or fungi do not have this matrix layer. The presence of matrix can cause failure of treatment with antimicrobial agents, relapse of infection, and increased mortality. ^13^ Penetration of antimicrobial agents can be complicated due to the formation of extracellular polysaccharides (EPS) which reduce the permeability of the biofilm thereby protecting microorganisms in the deepest layers of the biofilm from antimicrobial agents, minor mechanical stress, and host immune response. ^14^^,^^15^ To determine the inhibitory effect of an antimicrobial agent on biofilm formation, it can be done using the Minimum Inhibitory Biofilm Concentration (MBIC), which is almost the same as the MIC. The difference between the two is that MBIC is defined as the lowest concentration of an antimicrobial agent at which there is no time-dependent increase in the average number of cells capable of surviving in the biofilm. Meanwhile, MIC is defined as the lowest concentration of an antimicrobial agent against planktonic microorganisms. ^13^

To prevent the accumulation of denture plaque, adequate and routine denture cleaning needs to be done. Denture cleaning can be done chemically using alkaline peroxide type denture cleaning agent. However, alkaline peroxide was found not to show stable biofilm cleaning efficacy with previous studies showing that alkaline peroxide was not effective in cleaning biofilm and was only effective in cleaning new plaque. ^16^^,^^17^ Therefore, it is necessary to develop a denture cleanser product in solution preparation with natural ingredients that have antimicrobial effects which can effectively clean denture plaque. One example of natural ingredient that can be used as an antimicrobial and antibiofilm agent is avocado seeds.

Avocados ( *Persea americana* Mill.) are one of the most popular types of fruit among Indonesian and are widely used as food ingredients (salads, sandwiches, cakes) and drinks (juice, ice cream), cosmetic ingredients, medicines and ornamental plants. ^18^ However, avocado seeds have no practical use and have not been utilized optimally so they tend to be an organic waste. ^19^ Avocado seed can actually be used as an antimicrobial agent because of the higher amounts of phytochemical components contained in avocado seed, namely flavonoids, tannins, saponins, and alkaloids, than in avocado skin and pulp, which are 64% in seed, 23% in skin, and 13% in pulp. ^20^^,^^21^ The inhibitory effects of avocado seed extract has been studied. Anggraini et al. (2017) studied the inhibition zone of avocado seed extract at concentrations of 10%, 20%, 40%, 80%, 100% on the growth of *C. albicans*, and found that the 10% concentration was the most effective concentration in inhibiting *C. albicans.* ^22^ Another study by Talib et al. (2018) tested the effectiveness of avocado seed extract in inhibiting *Streptococcus mutans* at concentrations of 2%, 4%, 6%, 8%, 10% and found that the most effective concentration was 10%. ^23^

However, most studies using avocado seed extract were carried out on planktonic bacteria or fungi, that is different from denture plaque in the patient’s oral cavity, which is a polymicrobial biofilm that tends to be more resistant to antimicrobial agents. Unlike planktonic bacteria and fungi, polymicrobial biofilm has a protective layer, extracellular polysaccharide (EPS) formation, which decrease the permeability of the biofilm from antimicrobial agents. Moreover, the various microorganisms in the biofilm also build a communication mechanism called quorum sensing which enables the alteration of virulence gene expressions, the production and utilization of varied nutritions effectively through metabolic consortium, and even the ability to slow their growth in the presence of antimicrobial agents in the biofilm thereby suggesting a better resistance of polymicrobial biofilms towards antimicrobial agents than planktonic microorganism’s resistance. ^14^^,^^15^^,^^24^ This can be seen in a study by Hamzah et al. (2019) who found an increase in the minimum biofilm inhibitory concentration of tannin in polymicrobial biofilms ( *Escherichia coli*, *Staphylococcus aureus*, *Pseudomonas aeruginosa*, and *Candida albicans*) when compared to the concentration in mono-species biofilms. The minimum biofilm inhibitory concentration of tannin in mid-phase polymicrobial biofilms (24 hours) is 1%, while the minimum inhibitory concentration of tannins in mono-species biofilms (24 hours) varies widely, namely *E. coli* (0.125%), *S. aureus* (0.5%), *P. aeruginosa* (0.25%), *C. albicans* (0.5%). ^24^ Hence, this study aimed to determine the effect of avocado seed extract ( *Persea americana* Mill.) with concentrations of 5%, 10%, 15%, 20% on denture-plaque microorganisms, which were *Candida albicans*, *Candida glabrata*, *Actinomyces odontolyticus*, *Streptococcus gordonii*, and *Staphylococcus aureus*, in the form of mono-species and polymicrobial biofilms on HPA resin through determining their minimum biofilm inhibitory concentration (MBIC _50_).

## Methods

### Study design

**Figure 1.**
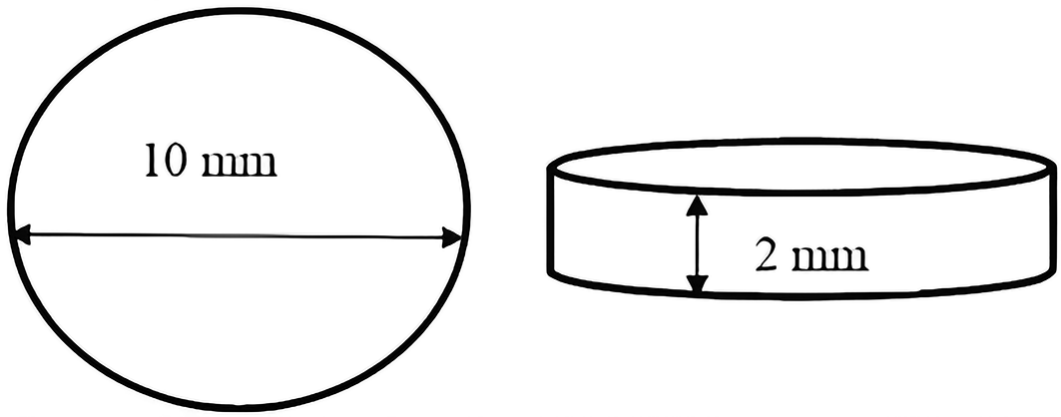
Shape and size of heat polymerized acrylic resin disc.

This research used in vitro experimental methods with post-test only control group design. The sample used in this research was HPA resin in the shape of a disc with a diameter of 10 mm and a thickness of 2 mm ( Figure 1). The number of samples used in this study was determined using Federer formula, hence the number of samples for each group was 4 samples. There were 6 treatment groups in this study which were avocado seed extract groups of 5%, 10%, 15%, 20%, as well as the positive control group (alkaline peroxide) and the negative control group (aquadest). As this research was conducted on mono-species biofilms: *Candida albicans*, *Candida glabrata*, *Streptococcus gordonii*, *Actinomyces odontolyticus*, *Staphylococcus aureus*, and on polymicrobial biofilm which is a combination of the five microorganisms, the final total amount sample that would be used in this research was 144 samples (n=144).

### Preparation of heat polymerized acrylic resin disc samples

A disc-shaped brass metal master models with a diameter of 10 mm and a thickness of 2 mm were made to be used as a research sample mould. The dental cuvette, which had been smeared with Vaseline, was poured with a type II blue dental stone gypsum mixture made with a ratio of 300 g of gypsum: 90 mL of water to fill the bottom cuvette while being shaken with a vibrator so that no bubbles were trapped in the mixture. The master models, which had been smeared with vaseline, were then placed in the dough in a cuvette, avoiding the surface of the master models being flat with the gypsum surface. The gypsum was left to harden for ± 30-45 minutes and then smeared with vaseline. The upper cuvette was attached to the lower cuvette and filled with the same gypsum mixture as described previously. After the plaster had hardened, the cuvette was opened and the master models were taken out to obtain a mould. ^25^

The surface of the mould was smeared thinly with cold mould seal and left for 10 minutes. The HPA resin mixture was prepared with a weight ratio of 2.5:1. When it reached the dough stage, the acrylic resin mixture was put into the mould, then covered with plastic cellophane along with the top cuvette. The cuvette was pressed slowly with a hydraulic press until the pressure reached 1000 psi. Excess dough was cleaned with dental lecron, then the cuvette was closed and pressed again with a pressure of 2200 psi. The cuvette was reopened and cleaned of excess acrylic resin mixture. The cuvette was closed again and locked with the cuvette bolts, then left for 30 minutes. The cuvette was inserted into a water bath filled with aquadest, then the temperature and time were set at 70°C for 90 minutes, then at 100°C for 30 minutes. After 30 minutes, the cuvette was left in the water bath until the water reached room temperature for the cuvette cooling process. The samples were removed from the cuvette, then the sharp parts and plaster residue were trimmed with a fraser bur and sand paper. ^25^

### Avocado seed extract procedure and tests

***Avocado sample***

Avocados were obtained from Berastagi, Karo Regency, North Sumatra Province, Indonesia. The avocado fruit used in this research has been determined by the Medanense Herbarium Plant Systematics Laboratory (MEDA) at the University of Sumatera Utara with letter number 1835/MEDA/2023.

***Avocado seed extraction procedure***

Avocado seeds were extracted using a maceration technique. The avocado seeds would be cut into slices which would be dried in a drying cabinet at a temperature of ±40°C for about 24 hours, then coarsely grounded and blended until they became a fine powder. The avocado seed powder was put into a vessel and poured with 70% ethanol solvent with a ratio of 1:10 (10 g: 100 mL), then stirred until evenly mixed and left for 1×24 hours protected from light while stirring periodically every 6 hours so that the solution was evenly mixed. The solution was filtered until macerate I was obtained and the remaining filtered dregs were subjected to a second maceration process. The results of macerate I and II would be mixed and transferred into a closed vessel, then left in a cool place protected from light for 2×24 hours. The extract was concentrated using a rotary evaporator at a temperature of ±50°C to evaporate the solvent until a thick extract was obtained. The thick extract was diluted with aquadest to be made into a concentration of 5%, 10%, 15%, 20%. ^26^

***Phytochemical examination and quantitative test of phytochemical compounds of avocado seed extract***

The thick extract of avocado seeds was sent to the Pharmaceutical Biology Laboratory, University of Sumatera Utara, Medan for phytochemical examination and quantitative testing of phytochemical compounds.

### Formation of denture plaques on heat polymerized acrylic resin disc samples

***Microorganisms and culture conditions***

The microorganisms used in this study were cultured and maintained under the following conditions. *Candida albicans* ATCC ^®^ 24433 ^TM^ and *Candida glabrata* ATCC ^®^ 90030 ^TM^ were each cultured on Sabouraud Dextrose Agar (SDA) with yeast nitrogen base supplemented with 100 mmol L ^−1^ glucose and cultured at 37°C under aerobic conditions for 24 hours. *Actinomyces odontolyticus* ATCC ^®^ 17982 ^TM^ was cultured on fastidious anaerobe agar with 5% (v/v) defibrinated bovine blood at 37°C under anaerobic conditions for 24 hours. *Streptococcus gordonii* ATCC ^®^ 10558 ^TM^ was cultured on blood agar with 5% (v/v) defibrinated bovine blood at 37°C under aerobic conditions for 24 hours. *Staphylococcus aureus* ATCC ^®^ 25923 ^TM^ was cultured on blood agar with 5% (v/v) defibrinated bovine blood and incubated at 37°C under aerobic conditions for 24 hours. ^27^

***Biofilm formation on heat polymerized acrylic resin disc***

HPA resin discs were first sterilized on both sides, each for 30 minutes, using ultraviolet germicidal irradiation technique. Sterile HPA resin discs were preconditioned for 24 hours by immersion in artificial saliva and after the immersion period, the HPA resin discs still immersed in artificial saliva were sterilized again. The density of the microorganism cultures must be adjusted using a densitometer following the 0.5 McFarland standard, namely 1.5 × 10 ^8^ CFU/mL for bacterial suspensions ( *A. odontolyticus*, *S. gordonii*, *S. aureus*) and 1.0 McFarland standard, namely 3.0 × 10 ^8^ CFU/mL for fungal suspensions ( *C. albicans* and *C. glabrata*). The preconditioned discs were then placed aseptically into 24-microplates, and 100 μL of standardized microorganisms were added to each surface of the disc. Biofilm preparations carried out were mono-species biofilms for each microorganism studied ( *C. albicans*, *C. glabrata*, *A. odontolyticus*, *S. gordonii*, *S. aureus*), and polymicrobial biofilms which were a combination of the five microorganisms studied, resulting in a total of 500 μL on each polymicrobial biofilms disc. Sterile Dulbecco’s Modified Eagle Medium (DMEM) (supplemented with 50 mmol L-1 L-glutamine per liter) was added to a final volume of 2 mL in each plate. Culture media discs in 24-wells microplates were shaken on an orbital shaker for 30 minutes to homogenize the media and culture solutions, then incubated at 37°C for 24 hours. ^27^

***Determination of minimum biofilm inhibitory concentration (MBIC _50_) using microtiter plate biofilm formation test***

HPA resin discs which had been grown by mono-species and polymicrobial biofilms would be treated with immersion in avocado seed extract of 5%, 10%, 15%, 20%, as well as positive control (alkaline peroxide) and negative control (aquadest) for 8 hours at room temperature. The discs were then cleaned with distilled water, then put into a test tube together with 5 mL of Mueller Hinton broth and each shaken with a vortex mixer for 1 minute. A total of 100 μL of test solution was taken from the dilution and added into 96-wells microplates with a repetition of three times. Microplates were incubated at 37°C for 24 hours. After incubation, the microplates were cleaned with distilled water and patted vigorously on a lab mat to remove as much distilled water as possible. As much as 125 μL of 1% crystal violet solution was added to each microplate to colour the formed biofilm and left for 15 minutes. The crystal violet solution was discarded, then cleaned with distilled water and patted hard on a lab mat. The stained biofilm plates were allowed to dry until the remaining water in the microplates evaporated, then 150 μL of 95% ethanol was added to each plate and left for 10 minutes. The absorption value (OD) reading was carried out with a microplate reader at a wavelength of 595 nm and the results were calculated using the percentage inhibition value formula of which Control OD was defined as negative control (aqaudest) absorption value and Sample OD was defined as test sample (avocado seed extracts and alkaline peroxide) absorption value. ^24^

Inhibition Value(%)=[(ControlOD−SampleOD)÷ControlOD]×100

The treated sample which had an inhibition value of at least 50% of biofilm formation could be considered as the Minimum Biofilm Inhibitory Concentration (MBIC _50_). ^24^

### Statistics analysis

Univariate analysis was carried out to determine the average (mean) and standard deviation of the inhibition values for immersion of heat polymerized acrylic resin discs in each group. The conversion of absorption value to inhibition value in percentage is counted using the percentage inhibition value formula that had been coded in Excel 2021 software. The normality test was carried out using the Shapiro-Wilk test (p>0.05) and the homogeneity test was carried out using the Levene test (p>0.05). Data analysis was carried out using one-way ANOVA, which could be accompanied by Welch ANOVA on non-homogeneous data, to determine the effect of treatment in each group. Data were analyzed with IBS SPSS Statistics (RRID: SCR_016479) v.22.0 software and presented in tabulation and graphic form as mean and standard deviation. Significant differences were defined at p<0.05.

### Scanning electron microscopy (SEM)

The SEM procedure was carried out at the USU Integrated Research Laboratory, Medan. HPA resin disc samples that had been preconditioned with artificial saliva were then grown with polymicrobial biofilm according to the previous biofilm formation procedure and given a soaking treatment in avocado seed extract. HPA resin disc samples that had biofilm grown on were cleaned with distilled water three times and fixed with 2.5% (w/v) glutaraldehyde in cacodylate buffer for about 6 hours. The wet sample was then coated with a thin layer of gold to make the sample conductive. Sample reading using SEM was carried out with a voltage of 5 kV.

### Ethical approval

The denture base subjects’ research was approved on 27 ^th^ February 2024 and performed according to the ethical standards by the Health Research Ethics Committee of the University of Sumatera Utara, Indonesia as stated in letter number 166/KEPK/USU/2024.

## Results

In this study, there were 6 treatment groups consisting of samples of HPA resin discs soaked in avocado seed extract 5%, 10%, 15%, 20%, as well as a positive control (alkaline peroxide) and a negative control (aquadest). The HPA resin disc samples were grown with mono-species biofilms of *C. albicans*, *C. glabrata*, *A. odontolyticus*, *S. gordonii*, *S. aureus* and polymicrobial biofilms so that the number of samples in this study was 144 samples (n=144).

### Determination of avocado fruit plants

The following are the results of avocado identification by the Medanense Herbarium, University of Sumatera Utara.

Kingdom: Plantae

Division: Spermatophyta

Class: Dicotyledoneae

Order: Laurales

Family: Lauraceae

Genus: Persea

Species: *Persea americana* Mill.

Local Name: Avocado Seed

### Phytochemical test results of avocado seed ethanol extract

The phytochemical test on the ethanol extract of avocado seeds was done using specific reagents to determine the presence of secondary metabolite compounds which were alkaloids, flavonoids, glycosides, saponin, tannin, triterpenoids/steroids ( Table 1).

**Table 1.** Phytochemical test of avocado seed ethanol extract.

No.	Secondary metabolites	Reagents	Results
1	Alkaloids	Dragendorff	+
		Bouchardat	+
		Mayer	+
2	Flavonoids	Mg Powder + Amyl Alcohol + HCl _p_	+
3	Glycosides	Molisch + H _2_SO _4_	+
4	Saponin	Hot water/shaken	+
5	Tannin	FeCl _3_	+
6	Triterpenoids/Steroids	Lieberman-Burchard	+

### Quantitative analysis results for phytochemical compounds of avocado seed ethanol extract

The secondary metabolite compounds existing in the avocado seed ethanol extract could be further assessed by doing a quantitative analysis to determine the amount of the secondary metabolites in the sample extract which were flavonoids, phenol, saponin, and alkaloids ( Table 2). The analysis showed the highest total amount of phenol and the lowest total amount of alkaloids. This might be caused by the extraction technique used in the research which was the maceration technique, a method that is very suitable for secondary metabolite compounds that are sensitive to heat, such as polyphenolic compounds, especially flavonoids. Extracting the avocado seed with maceration technique can cause the discovery of high levels of flavonoids and polyphenols. ^26^

**Table 2.** Quantitative analysis for phytochemical compounds of avocado seed ethanol extract.

No.	Analysis	Total	Unit
1	Total Flavonoids	4,0888	mgQE/g extract
2	Total Phenol	66,8157	mgGAE/g extract
3	Total Saponin	1,59	%
4	Total Alkaloids	1,22	%

### The MBIC _50_ determination of avocado seed extract and its effect on the mono-species biofilms of *C. albicans*, *C. glabrata*, *A. odontolyticus*, *S. gordonii*, *S. aureus*

Each sample in each group was repeated three times to obtain three absorption values (OD) which then using univariate analysis, the mean and standard deviation were obtained. The obtained absorption value was calculated using the percentage inhibition value formula with an inhibition value of 50% as a parameter for determining MBIC _50_ ( Table 3). Based on calculations, MBIC _50_ of avocado seed extract in mono-species *C. albicans* biofilm was 5% avocado seed extract. MBIC _50_ avocado seed extract in mono-species *C. glabrata* biofilm was 5% avocado seed extract. MBIC _50_ avocado seed extract in mono-species *A. odontolyticus* biofilm was 15% avocado seed extract. MBIC _50_ avocado seed extract in mono-species *S. gordonii* biofilm was 15% avocado seed extract. MBIC _50_ avocado seed extract in mono-species *S. aureus* biofilms was 10% avocado seed extract.

**Table 3.** Inhibition value of the mono-species biofilm growth soaked in avocado seed extract and alkaline peroxide.

Mono-species	Inhibition value (%)					
	Avocado Seed Extract				Positive control	Negative control
	5%	10%	15%	20%		
*Candida albicans*	76.09 ± 3.21 *	69.33 ± 8.57	77.80 ± 6.58	63.29 ± 7.85	66.43 ± 28.29	0.00 ± 0.00
*Candida glabrata*	52.41 ± 9.64 *	59.99 ± 7.55	58.55 ± 12.52	60.75 ± 15.80	67.24 ± 15.41	0.00 ± 0.00
*Actinomyces odontolyticus*	29.42 ± 5.21	40.50 ± 5.65	50.88 ± 7.31 *	52.58 ± 15.38	54.25 ± 13.87	0.00 ± 0.00
*Streptococcus gordonii*	38.52 ± 5.04	42.95 ± 3.61	51.16 ± 4.74 *	52.79 ± 4.26	69.79 ± 22.20	0.00 ± 0.00
*Staphylococcus aureus*	45.30 ± 4.05	51.63 ± 5.05 *	52.79 ± 5.05	56.15 ± 4.98	61.38 ± 8.54	0.00 ± 0.00

* = MBIC _50_, inhibition values are in mean and standard deviation.

The sample was tested for normality and a value of p>0.05 was obtained, hence the data were normally distributed. Then, a homogeneity test was carried out by which the mono-species *C. albicans* biofilm sample obtained a value of p<0.001 (p<0.05), the mono-species *A. odontolyticus* biofilm sample obtained a value of p=0.002 (p<0.05), and the mono-species *S. gordonii* biofilm sample obtained a value of p≤0.001 (p<0.05) so the data were not homogeneous, while the mono-species *C. glabrata* biofilm sample obtained a value of p=0.054 (p>0.05) and the mono-species *S. aureus* biofilm sample obtained a value of p=0.116 (p>0.05) so the data were homogeneous. Data which was normally distributed and homogeneous was tested using one-way ANOVA and data which was normally distributed but not homogeneous was analysed using Welch ANOVA. This study found that mono-species biofilm samples of *C. albicans, C. glabrata, A. odontolyticus, S. gordonii, S. aureus* obtained a value of p≤0.001 (p<0.05) which indicated a significant effect of 5%, 10%, 15%, 20% avocado seed extract in inhibiting mono-species biofilms of *C. albicans, C. glabrata, A. odontolyticus, S. gordonii, S. aureus* ( Table 4).

**Table 4.** One-way ANOVA and Welch ANOVA of the treatment groups against mono-species biofilms.

Treatment groups	Absorption values	p
**Mono-species *C. albicans* Biofilm**		
Avocado Seed Extract of 5%	0.2949 ± 0.0397	<0.001 *
Avocado Seed Extract of 10%	0.3782 ± 0.1057	
Avocado Seed Extract of 15%	0.2738 ± 0.0812	
Avocado Seed Extract of 20%	0.4527 ± 0.0968	
Positive control (+) Alkaline Peroxide	0.4140 ± 0.3489	
Negative control (-) Aquadest	1.2332 ± 0.1059	
**Mono-species *C. glabrata* Biofilm**		
Avocado Seed Extract of 5%	1.0400 ± 0.2107	<0.001 *
Avocado Seed Extract of 10%	0.8743 ± 0.1651	
Avocado Seed Extract of 15%	0.9057 ± 0.2735	
Avocado Seed Extract of 20%	0.8576 ± 0.3452	
Positive control (+) Alkaline Peroxide	0.7160 ± 0.3368	
Negative control (-) Aquadest	2.0627 ± 0.2138	
**Mono-species *A. odontolyticus* Biofilm**		
Avocado Seed Extract of 5%	1.4803 ± 0.1093	<0.001 *
Avocado Seed Extract of 10%	1.2479 ± 0.1185	
Avocado Seed Extract of 15%	1.0302 ± 0.1534	
Avocado Seed Extract of 20%	0.9946 ± 0.3225	
Positive control (+) Alkaline Peroxide	0.9595 ± 0.2909	
Negative control (-) Aquadest	2.0972 ± 0.1524	
**Mono-species *S. gordonii* Biofilm**		
Avocado Seed Extract of 5%	1.0829 ± 0.0887	<0.001 *
Avocado Seed Extract of 10%	1.0048 ± 0.0635	
Avocado Seed Extract of 15%	0.8602 ± 0.0834	
Avocado Seed Extract of 20%	0.8316 ± 0.0751	
Positive control (+) Alkaline Peroxide	0.5322 ± 0.3910	
Negative control (-) Aquadest	1.7614 ± 0.0895	
**Mono-species *S. aureus* Biofilm**		
Avocado Seed Extract of 5%	0.9012 ± 0.0666	<0.001 *
Avocado Seed Extract of 10%	0.7968 ± 0.0832	
Avocado Seed Extract of 15%	0.7779 ± 0.0832	
Avocado Seed Extract of 20%	0.7225 ± 0.0820	
Positive control (+) Alkaline Peroxide	0.6362 ± 0.1407	
Negative control (-) Aquadest	1.6475 ± 0.0884	

* Significant, absorption values are in mean and standard deviation.

### The MBIC _50_ determination of avocado seed extract and its effect on polymicrobial biofilm

In this study, three absorption values (OD) of polymicrobial biofilm samples were obtained from which the mean and standard deviation were obtained using univariate analysis. Using the percentage inhibition value formula, the inhibition values were obtained for each group of avocado seed extract of 5%, 10%, 15%, 20%, and the positive control (alkaline peroxide) where the 50% inhibition value was set as a parameter for determining MBIC _50_ ( Table 5). Hence, the MBIC _50_ avocado seed extract in polymicrobial biofilm was 20% avocado seed extract.

**Table 5.** Inhibition value of the polymicrobial biofilm growth soaked in avocado seed extract and alkaline peroxide.

Polymicrobial biofilm	Inhibition value (%)					
	Avocado Seed Extract				Positive control	Negative control
	5%	10%	15%	20%		
	31.94 ± 11.53	36.32 ± 12.24	45.36 ± 5.70	50.81 ± 8.32 *	63.10 ± 28.26	0.00 ± 0.00

* = MBIC _50_, inhibition values are in mean and standard deviation.

The sample was tested for normality and a value of p >0.05 was obtained, hence the data was normally distributed. Then, a homogeneity test was carried out by which the polymicrobial biofilm samples obtained a value of p=0.006 (p<0.05) so that the data was not homogeneous. Data that were normally distributed but not homogeneous were analysed using the Welch ANOVA. This study found that the polymicrobial samples obtained a value of p≤0.001 (p<0.05) which indicated a significant effect of soaking in 5%, 10%, 15%, 20% avocado seed extract in inhibiting polymicrobial biofilm ( Table 6).

**Table 6.** Welch ANOVA of the treatment groups against polymicrobial biofilm.

Kelompok	Absorption Values of Polymicrobial Biofilm	p
Avocado Seed Extract of 5%	1.3573 ± 0.2300	<0.001 *
Avocado Seed Extract of 10%	1.2699 ± 0.2442	
Avocado Seed Extract of 15%	1.0897 ± 0.1137	
Avocado Seed Extract of 20%	0.9810 ± 0.1658	
Positive control (+) Alkaline Peroxide	0.7359 ± 0.5637	
Negative control (-) Aquadest	1.9942 ± 0.6417	

* Significant, absorption values are in mean and standard deviation.

### Scanning electron microscopy (SEM) results of polymicrobial biofilm on hpa resin discs soaked in avocado seed extract

**Figure 2.**
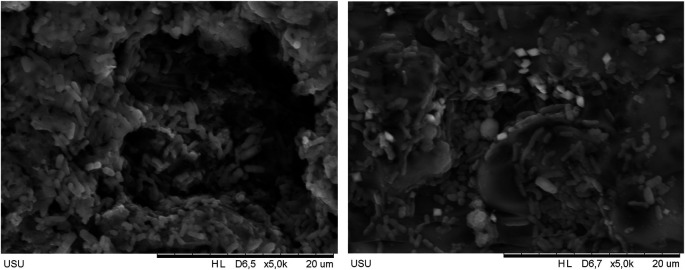
SEM results of 5% avocado seed extract (left) and 15% avocado seed extract (right).

Based on the Integrated Laboratory Test Results Report of the University of Sumatera Utara with the number 113/UN5.4.6.K/KPM/2024, the results of SEM tests carried out on HPA resin discs with polymicrobial biofilm which had been soaked with avocado seed extract could be detected and clearly seen in Figure 2 above. SEM results showed that there were microorganisms growing on the HPA resin disc. The soaking in 5% avocado seed extract showed a denser formation of biofilm compared to soaking in 15% avocado seed extract.

## Discussion

Denture plaque is not the same as dental plaque, although the microbial composition of denture plaque is influenced to a certain extent by dental plaque because the microbiota on the denture surface and the tooth surface originates from the same oral cavity. However, Fujinami et al. (2021) and O’Donnell et al. (2015) found lower diversity in denture plaque compared to dental plaque which may be caused by differences in the surface on which it grows. ^8^^,^^28^ Low diversity in denture plaque, and the denture environment in the underlying tissue which is low in oxygen levels and saliva flow, as well as denture surface characteristics in the form of porosity and non-specific factors such as hydrophobicity, van der Waals forces, Brownian motion forces, and electrostatic interactions support the adhesion and growth of *Candida* spp. to colonize denture surfaces and form co-aggregates with bacteria to form complex microbial communities. ^8^^,^^26^^,^^29^

### The MBIC _50_ of avocado seed extract and its effect on the mono-species biofilms of *C. albicans*, *C. glabrata*, *A. odontolyticus*, *S. gordonii*, *S. aureus*

*Candida albicans* has the pathogenic ability in the form of morphogenesis, which is the ability to transition *C. albicans* from a unicellular yeast form to a pathogenic filament form (pseudohyphae or hyphae) reversibly. ^30^^,^^31^ *C. albicans* yeast cells will adhere to the denture surface via A1s1-8p adhesin, then proliferate to form microcolonies which become the basal layer of the biofilm and produce extracellular matrix (ECM). As the biofilm matures, there is an increase in biomass with the presence of yeast cells, hyphae and pseudohyphae encapsulated in the extracellular matrix. These hyphae are fundamental and important components in supporting the structural integrity of the biofilm and provide a means of attachment for additional yeast cells, pseudohyphae, other hyphae, and bacteria due to their ability to express specific adhesins such as Hwp1p and Hyr1p. ^15^ These hyphae are also capable of damaging epithelial cells and destabilizing membranes through induced calcium ion influx and release of lactate dehydrogenase. ^9^ In this study, the MBIC _50_ of avocado seed extract in mono-species *C. albicans* biofilm was 5% avocado seed extract with an inhibition value of 76.09 ± 3.21%. This is in accordance with previous research by Wulandari et al. (2023) who tested the effect of avocado seed extract on *C. albicans* biofilms. Using the inhibition percentage formula, the lowest concentration of avocado seed extract tested, which is a concentration of 3.13%, was able to inhibit *C. albicans* biofilms incubated for 24 hours by 75.37%. ^32^ These results indicated that avocado seed extract was effective in inhibiting *C. albicans*, considering that the lowest concentration of avocado seed extract had already shown a greater inhibition value by roughly 25% than the standard inhibition value.

*Candida glabrata* is the second most frequently isolated cause of candidiasis and is often found together with *C. albicans* in the form of co-isolation in which *C. glabrata* budding yeast is found attached to C. albicans hyphae. ^33^ There was still little to none research done on the effect of avocado seed extract on *C. glabrata.* This study found that the MBIC _50_ of avocado seed extract in mono-species *C. glabrata* biofilm was 5% avocado seed extract with an inhibition value of 52.41 ± 9.64%. When compared with the *C. albicans* inhibition value, there was a decrease in the biofilm inhibition activity of avocado seed extract against *C. glabrata.* This is due to the higher antifungal resistance in *C. glabrata* than in *C. albicans*, and the rapid ability of *C. glabrata* to develop resistance to currently used antifungal agents. ^34^ Farahyar et al. (2016) found that *C. glabrata* had Candida drug-resistant (CgCDR) genes CgCDR1 and CgCDR2, and Fatty Acid Activator 1 (FAA1) which was positively regulated twice as much in resistant strains. ^35^ Yu et al. (2018) found that another factor that played an important role in the antifungal tolerance and cell wall integrity of *C. glabrata* is ADA2 which was mediated by the ERG6 gene. ^36^

Bacteria are thought to play an important role in the formation of denture plaque considering that denture plaque can contain 10 ^11^ microbes per milligram. ^8^ *Actinomyces* is a genus commonly found in denture plaque with a large proportion which can be caused by the ability of *C. albicans* biofilms to provide a positive anaerobic environment to some anaerobic bacteria so that *Actinomyces* which is an anaerobic bacteria can easily grow in oxygen-rich areas. ^10^^,^^31^ However, the clinical significance of *Actinomyces* spp. still needs to be proven and the available data regarding the antimicrobial susceptibility of *Actinomyces* is still limited with the susceptibility method which has not been standardized. In this study, the MBIC _50_ of avocado seed extract against the mono-species *Actinomyces odontolyticus* biofilm was high, namely 15% avocado seed extract with an inhibition value of 50.88 ± 7.31%. This can be explained by several studies which had found the existence of antimicrobial agent resistance or antibiotic resistance in *A. odontolyticus.* Wolff et al. (2022) found an *A. odontolyticus* isolate that showed multi-drug resistance (MDR) to benzylpenicillin, meropenem, moxifloxacin, and daptomycin. ^37^ Steininger et al. (2016) tested the susceptibility of *Actinomyces* spp. taken from 387 patients over a 7-year period and found that *Actinomyces* spp. was susceptible to β-lactam antimicrobial agents with and without β-lactamase inhibitors and there was an *A. odontolyticus* isolate that was resistant to tetracycline. ^38^

Shi et al. (2016) found that *S. gordonii* colonized denture teeth in healthy denture users at a significantly higher rate. ^11^ In this study, the MBIC _50_ of avocado seed extract in mono-species *S. gordonii* biofilm was 15% avocado seed extract with an inhibition value of 51.16 ± 4.74%. There was still no research done on the effect of avocado seed extract on *S. gordonii*, but most researches on *S. mutans* had been carried out, where these researches focused on treating caries and dental plaque rather than denture plaque. Calosa et al. (2023) found that the minimum inhibitory level of avocado seed extract against S. mutans as seen from the sample absorbance was 12.5%. ^39^ *S. gordonii* usually competes with *S. mutans* where *S. gordonii* metabolically produces hydrogen peroxide which is able to inhibit the growth of *S. mutans*, and produces alkaline ammonia which is able to mitigate acidity on the tooth surface. The presence of *S. mutans* in plaque is strongly and positively associated with caries while *S. gordonii* is negatively associated with caries. ^40^ Considering the antagonistic relationship between *S. mutans* and *S. gordonii*, the presence of *S. gordonii* in denture plaque minimizes the presence of *S. mutans.* ^39^ Further research into the effects of avocado seeds on *S. gordonii* needs to be carried out.

*S. aureus* is often associated with higher amount in the elderly, seriously ill patients, individuals with low salivary secretion, and denture wearers. ^41^ *S. aureus* is also commonly found in patients with oral infections associated with *Candida albicans*, such as denture stomatitis and angular cheilitis, due to the nature of *S. aureus* which tends to attach more easily to the hyphal phase of *C albicans* compared to abiotic surfaces. ^42^ In this study, the MBIC _50_ of avocado seed extract in mono-species *S. aureus* biofilm was 10% avocado seed extract with a value of inhibition was 51.63 ± 5.05%. Research on the effect of avocado seed extract on *S. aureus* that has been carried out has found the Minimum Inhibitory Concentration (MIC) of avocado seed extract, but not the MBIC. Santosa et al. (2019) using the zone of inhibition test concluded that avocado seed extract was effective in inhibiting multi-resistant *S. aureus* at a concentration of 6.25%. ^43^

This study found a significant effect of soaking in avocado seed extract ( *Persea americana* Mill.) concentrations of 5%, 10%, 15%, 20%, as well as the positive control of alkaline peroxide in inhibiting the growth of denture plaque microorganisms on HPA resin discs in the form of *C. albicans*, *C. glabrata, A. odontolyticus, S. gordonii*, and *S. aureus* mono-species biofilms, each with a value of p≤0.001 (p<0.05). If the inhibition value of avocado seed extract was compared with the inhibition value of the positive control alkaline peroxide, only the MBIC _50_ inhibition value of avocado seed extract on mono-species *C. albicans* biofilm (76.09 ± 3.21%) was found to be higher than the inhibition value of the positive control (66, 43 ± 28.29%). In other mono-species biofilms, such as C. glabrata, A. odontolyticus, S. gordonii, S. aureus, the inhibition value of avocado seed extract was lower than the inhibition value of the positive control. This can be explained by the findings of Morelli et al. (2023), which stated that effervescent tablets showed good antimicrobial activity against *C. glabrata, S. mutans*, and *S. aureus* on a cobalt-chromium surface. However, none of these peroxide-based solutions showed a reduction in *C. albicans* biofilms or substantially eliminated aggregated biofilms. ^44^

### The MBIC _50_ of avocado seed extract and its effect on polymicrobial biofilm

From the research results, the MBIC _50_ of avocado seed extract against polymicrobial biofilm in this study was 20% avocado seed extract with an inhibition value of 50.81 ± 8.32%. When compared with MBIC _50_ in mono-species biofilms, it was found that polymicrobial biofilm required a higher concentration of avocado seed extract. This is in accordance with research results which state that polymicrobial biofilms have higher resistance to antimicrobial agents compared to mono-species biofilms. O’Brien et al. (2022) who tested three clinically relevant antimicrobial agents namely colistin, fusidic acid, and fluconazole against polymicrobial populations containing *P. aeruginosa, S. aureus*, and *C. albicans* found a higher antimicrobial agent resistance in polymicrobial biofilm compared to mono-species biofilms. These researchers found that there was a decrease in antimicrobial activity against target microorganisms in polymicrobial cultures compared to mono-species cultures. ^45^ However, Kart et al. (2014) stated that polymicrobial biofilms did not always have higher resistance compared to mono-species biofilms as its susceptibility to antimicrobial agents depends on the nature of the microbial species present and the disinfectant used. ^46^

In polymicrobial biofilms, the interactions between microbes are very complex, some of which include cooperative and antagonistic interactions. Synergism between species in polymicrobial biofilms can produce effects on growth enhancement, antimicrobial resistance, virulence, and greater exopolysaccharide production compared to individual species alone. ^47^ *C. albicans* and *C. glabrata* are often found together in the form of co-isolates that cause increased pathogenicity of both species. ^48^ This is due to the ability of *C. albicans* to damage host tissue which can be exploited by *C. glabrata* to reach deeper tissues. *C. glabrata* itself has very high antifungal resistance capabilities, and is able to modify the maturation of macrophage phagosomes so that they can hide inside macrophages from the host immune system so *C. glabrata* can produce infections that are much more severe and require quite complicated treatment. ^49^ Other microorganisms that were found to have a very synergistic interaction were *S. gordonii* and *C. albicans.* *S. gordonii* was found to be capable of promoting filamentation and increasing fungal biofilm formation. Higher biomass was also found in polymicrobial biofilms formed by *C. albicans* and *S. gordonii.* ^50^ Diaz et al. (2014) showed an increase in the ability of oral streptococci to form biofilms on abiotic surfaces in the presence of *C. albicans.* ^51^ This is caused by C. albicans adhesins which facilitate the interaction of bacterial species, such as Als1p, Als2p, Als3p, Hwp1p. ^30^ On the other hand, these bacteria is able to influence the local environment of C. albicans by altering nutrient supply and carbon dioxide levels thereby favouring C. albicans’ hyphal transition and virulence. ^51^ The interaction of the two species causes increased resistance to antimicrobial agents. ^50^ The relationship between *S. aureus* and *C. albicans* has also been studied extensively where *C. albicans* can increase *S. aureus* resistance to vancomycin by 100-fold due to the production of the cell wall component β-1,3-glucan. These compounds were identified as matrix constituents that provide bacteria with increased drug tolerance. In addition, the production of farnesol and prostaglandin E2 by C. albicans can increase S. aureus biofilm formation. ^52^

Antagonistic interactions are a type of competitive interaction where one species will inhibit the growth of another species by producing a variety of secondary metabolites that can inhibit or kill competing species so that the biofilm architecture can be disrupted. ^47^ Guo et al. (2015) found an inhibitory effect of *A. odontolyticus* on proliferation, adhesion, metabolic enzyme activity, hypha formation, and biofilm development of *C. albicans.* Actinomyces was found to produce many metabolites with antifungal activity, including lincomycin and geldanamycin. ^53^ However, another study by Morse et al. (2019) showed opposite results and found that polymicrobial biofilms of *S. sanguinis, S. gordonii, A. odontolyticus,* and *A. viscosus* were able to increase the number of C. albicans hyphae. ^27^

In this study, there was a significant effect of soaking in avocado seed extract ( *Persea americana* Mill.) concentrations of 5%, 10%, 15%, 20%, as well as the positive control of alkaline peroxide in inhibiting the growth of polymicrobial biofilm on HPA resin discs with a value of p≤0.001 (p<0.05). The results of this analysis were also supported by SEM results which showed a much sparse biofilm on HPA resin discs soaked in 15% avocado seed compared to those soaked in 5% avocado seed extract. This showed that avocado seed extract had the ability to damage the mucus layer of polymicrobial biofilms. Polymicrobial biofilms are highly structured associations of microorganisms encased in an extracellular matrix (ECM) which attached to biotic or abiotic surfaces. One of the advantages of biofilms to the microorganisms within them is the presence of collective recalcitrant which is defined as the ability of pathogenic biofilms to survive in the presence of high concentrations of antibiotics. Cells in biofilms were found to be 10-1000 times more resistant to various antimicrobial agents than their planktonic forms. ^54^ Polymicrobial biofilms were found to have tolerance to antimicrobial agents and increased virulence due to an extracellular matrix (ECM) containing abundant extracellular polymeric substances (EPS) to protect all microbial cells from various dangers. ^55^ The presence of extracellular matrix (ECM) can influence pH, oxygen concentration, and nutrient availability in the deepest layers of the biofilm. In addition, ECM can limit the penetration of antimicrobial agents and cause the accumulation of antibiotic-degrading enzymes. ^46^ Therefore, increasing the permeability of polymicrobial biofilms is one of the targets of antimicrobial agents to inhibit the microorganisms within them.

In the phytochemical tests that have been carried out, flavonoid, tannin, alkaloid, saponin, triterpenoid and polyphenol class compounds were found present in avocado seed extract. Followed by quantitative tests of phytochemical compounds, it was found that the total flavonoid content in avocado seed extract was 4.0888 mgQE/g, the total phenol content was 66.8157 mgGAE/g, the total alkaloid content was 1.22%, and the total saponin content was 1. 59%. The result is aligned with the study of Vinha et al. (2013) which found higher levels of flavonoids and total phenolics in avocado seeds compared to avocado flesh and skin. ^56^ Flavonoids and tannins are a family of polyphenolic components that are widely distributed in Kingdom Plantae. ^57^ Flavonoids were found to have an antibiofilm effect by penetrating the biofilm layer and inhibiting bacterial growth and attachment. surface. The presence of hydrophilic parts of the chemical structure of flavonoids, including glycoside and hydroxy groups, could increase penetration of the biofilm structure and increase antibiofilm activity. ^29^ Matilla-Cuenca et al. (2020) found that the antibiofilm activity of flavonoids which could inhibit *S. aureus* biofilm formation was specifically mediated by Bap. ^58^ Tannins were also found to influence the gene expression of virulent factors such as biofilm, enzymes, adhesins, motility and toxins, and act as quorum sensing inhibitors. ^59^ Villanueva et al. (2023) found that all unmodified natural tannins had broad spectrum activity due to their ability to exhibit very significant anti-biofilm activity against Gram-positive and Gram-negative bacteria at least at a concentration of 150 mg/L. ^60^

Alkaloids have been found to damage bacterial cell membranes, inhibit efflux pumps, inhibit ATP synthesis which affects the metabolic processes of microorganisms, damage DNA/RNA molecules or inhibit DNA thereby preventing the expression of virulent genes, and inhibit FtsZ protein synthesis by participating in the diaphragm formation and forming a ring structure in division sites to control the division process and growth of bacterial cells. ^61^ Saponin can reduce the surface tension of bacterial cell walls and damage cell permeability so that saponin can diffuse into the cell and bind to the cytoplasmic membrane which can lead to cell lysis. ^59^ This activity can facilitate the influx of antimicrobial agents to the deeper layers of the polymicrobial biofilm. Brahim et al. (2015) found that the combination of saponin extract with fluconazole showed good synergism against *C. albicans, C. parapsolosis, C. krusei*, and *C. glabrata.* ^62^ Monte et al. (2014) showed the potential of saponins in controlling the shape of plankton and biofilms of *E. coli* and *S. aureus.* ^63^ Triterpenoids with more polar groups such as hydroxyl, carboxyl and carbonyl have anti-biofilm activity due to their hydrophilic nature so they are able to penetrate the exopolysaccharide polymeric matrix in bacterial biofilms and has an effect on bacterial cells in the biofilm, and shows anti-quorum sensing activity. ^64^

The inhibition value of the positive control alkaline peroxide against polymicrobial biofilm was found to be higher (63.10 ± 28.26%) than the MBIC _50_ inhibition value of 20% avocado seed extract (50.81 ± 8.32%). This shows that alkaline peroxide has a good anti-biofilm effect. Kaypetch et al. (2023) found that acrylic resin soaked in alkaline peroxide for more than 3 hours could efficiently penetrate and inhibit multispecies denture biofilm with an effect comparable to immersion in 0.5% NaClO for 10 minutes. ^65^ Research by Lucena-Ferreira et al. (2013) found that daily use of alkaline peroxide could improve denture cleanliness by reducing total microorganisms and total *Streptococcus*, but had no effect on the *Candida* spp. population. ^66^ However, MBIC _50_ avocado seed extract has been declared effective in inhibiting polymicrobial biofilm with an inhibition value exceeding 50% so that 20% avocado seed extract has the potential to be applied clinically as a natural denture cleanser. As avocado seed is a natural waste product which can cause significant environmental consequences if not managed properly, repurposing it as a composition of natural denture cleanser might not only resolve its environmental issues but also potentially lower the cost of the current denture cleanser and might show a much better biocompatibility with denture users than the current ones. Not to mention, avocado seed has larger amounts of antioxidants compared to its pulp and skin, which made avocado seed as one of the natural products that needs to be further studied.

### Study limitations

Several limitations have been found in this study. First, the diversity and composition of microorganisms in the polymicrobial biofilm in this study is a broad generalization of the diversity and composition of denture plaque in denture wearers. However, the microorganisms studied are the more commonly found denture plaque microorganisms and since avocado seed extract could inhibit these microorganisms, it shows that avocado seed could be one of the natural wastes that can be further studied and potentially used as natural denture cleaning agent. Second, the research was carried out in vitro, which means that all research variables were under the control of the researcher, which cannot be used to represent the condition of the oral cavity in patients using dentures that can be influenced by factors such as age, gender, habits, and so on. Hence, further studies involving humans or denture users should be conducted. Third, this research can only tell how much of the biofilm biomass that can be inhibited with avocado seed extract, but cannot know which microorganisms are inhibited in the polymicrobial biofilm. Nonetheless, considering the different diversity and composition of each denture users, it might not be as crucial to know the type of microorganisms inhibited as the reduction of the denture plaque itself. Lastly, further studies need to be done in order to determine which phytochemical component of the avocado seed extract that are effective in inhibiting denture plaque microorganisms and to gain more comparison data.

#### Ethical considerations

This study did not include any human participants or animal.

## Data Availability

Figshare: Avocado Seed Extract on Inhibiting Mono-species and Polymicrobial Biofilm. https://doi.org/10.6084/m9.figshare.25996006. ^67^ This project contains the following underlying data:
• Ethical Clearance No. 166KEPKUSU2024. pdf • Determination of Avocado Fruit Plants. pdf • Phytochemical Test Results of Avocado Seed Ethanol Extract. pdf • Quantitative Analysis for Phytochemical Compounds. pdf • Research Data of Mono-species C. albicans Biofilm. docx • Research Data of Mono-species C. glabrata Biofilm. docx • Research Data of Mono-species A. odontolyticus Biofilm. docx • Research Data of Mono-species S. gordonii Biofilm. docx • Research Data of Mono-species S.aureus Biofilm. docx • Research Data of Polymcrobial Biofilm. docx Data are available under the terms of the Creative Commons Attribution 4.0 International license (CC-BY 4.0)

## References

[1] Ferro KJ : The glossary of prosthodontic terms (GPT-9). *J. Prosthet. Dent.* 2017;117(5S):C1–e105. 10.1016/j.prosdent.2016.12.001 28418832

[2] Kangsudarmanto Y Rachmadi P Arya WI : Perbandingan perubahan warna heat cured acrylic basis gigi tiruan yang direndam dalam klorhesidin dan effervescent ( *alkaline peroxide*). *DENTINO.* 2014;2(2):205–9. 2337-5310.

[3] Fadriyanti O Alamsyah Y Rabianti D : Evaluasi pemakaian denture adhesive pada gigi tiruan lengkap resin akrilik: Scoping review. *Menara Ilmu.* 2022;16(2):55–62. 10.31869/mi.v16i2.3289

[4] Zarb G Hobkirk JA Eckert SE : *Prosthodontic treatment for edentulous patients.* 13th ed. St. Louis: Elsevier Mosby;2013;133–134.

[5] Bohra PK Ganesh PR Reddy MM : Colour stability of heat and cold cure acrylic resins. *J. Clin. Diagn. Res.* 2015;9(1):ZC12–ZC15. 10.7860/JCDR/2015/11620.5400 25738078 PMC4347169

[6] Rashid AA : Temperature effect on the hardness of different types of resin denture base materials. *MDJ.* 2013;10(1):69–76. 10.32828/mdj.v10i1.186

[7] Jain V Babu J Ahuja S : Comparison of fungal biofilm formation on three contemporary denture base materials. *Int. J. Exp. Dent.* 2015;4(2):104–108. 10.5005/jp-journals-10029-1106

[8] O’Donnell LE Robertson D Nile CJ : The oral microbiome of denture wearers is influenced by levels of natural dentition. *PLoS One.* 2015;10(9):e0137717. 10.1371/journal.pone.0137717 26368937 PMC4569385

[9] Patel M : Oral cavity and *Candida albicans*: Colonisation to the development of infection. *Pathogens.* 2022;11(335):6–8. 10.3390/pathogens110303355 PMC8953496 35335659

[10] Coulthwaite L Verran J : Potential pathogenic aspects of denture plaque. *Br. J. Biomed. Sci.* 2007;64(4):180–189. 10.1080/09674845.2007.11732784 18236742

[11] Shi B Wu T McLean J : The denture-associated oral microbiome in health and stomatitis. *mSphere.* 2016;1(6):e00215–e00216. 10.1128/mSphere.00215-16 28066812 PMC5196032

[12] Morse DJ Wilson MJ Wei X : Denture-associated biofilm infection in three-dimensional oral mucosal tissue models. *J. Med. Microbiol.* 2018;67:364–375. 10.1099/jmm.0.000677 29458673 PMC5882079

[13] Thieme L Hartung A Tramm K : MBEC versus MBIC: The lack of differentiation between biofilm reducing and inhibitory effects as a current problem in biofilm methodology. *Biol. Proced. Online.* 2019;21(18):15–18. 10.1186/s12575-019-0106-0 31528123 PMC6743098

[14] Kumari KS Dash P Subudhi E : Antimicrobial resistance: A dentists’ prospective. *Indian J. Med. Forensic Med. Toxicol.* 2020;14(4):8456–8460. 10.37506/ijfmt.v14i4.13018

[15] Ponde NO Lortal L Ramage G : *Candida albicans* biofilms and polymicrobial interactions. *Crit. Rev. Microbiol.* 2021;47(1):91–111. 10.1080/1040841X.2020.1843400 33482069 PMC7903066

[16] Peracini A Regis RR Souza RF : Alkaline peroxides versus sodium hypochlorite for removing denture biofilm: A crossover randomized trial. *Braz. Dent. J.* 2016;27(6):700–704. 10.1590/0103-6440201600913 27982182

[17] Oussama M Ahmad H : Materials and methods for cleaning dentures- A review. *Int. J. Dent. Clin.* 2014;6(2):19–20. Reference Source

[18] Lestari R Sukamto LA Aprilianti P : Selection of avocado plants based on fruit characters, fat content, and continual harvest along the year in west java-indonesia. *Int. J. Adv. Sci. Eng. Inf. Technol.* 2016;6(1):77–83. 10.18517/ijaseit.6.1.621

[19] Domínguez MP Araus K Bonert P : The avocado and its waste: An approach of fuel potential application. Lefebvre G Jiménez E Cabañas B , editors. *Environment, energy and climate change II: Energies from new resources and the climate change.* Switzerland: Springer Cham;2016;199–223. 10.1007/698_2014_291

[20] Setyawan HY Sukardi S Puriwangi CA : Phytochemicals properties of avocado seeds: A review. *IOP Conf. Ser.: Earth Environ. Sci.* 2021;733(1):012090–012097. 10.1088/1755-1315/733/1/012090

[21] Bahru TB Tadele ZH Ajebe EG : A review on avocado seed: Functionality, composition, antioxidant and antimicrobial properties. *Chem. Sci. Int. J.* 2019;27(2):1–10. 10.9734/CSJI/2019/v27i230112

[22] Anggraini V Masfufatun M : Efektivitas kombinasi ekstrak daun sirih merah ( *Piper crocatum*) dan ekstrak biji alpukat ( *Persea americana*) dalam menghambat pertumbuhan *Candida albicans.* *Jurnal Kimia Riset.* 2017;2(2):86–92. 10.20473/jkr.v2i2.6196

[23] Thalib B Nahar CL : Efektivitas antibakteri ekstrak biji alpukat ( *Persea americana* Mill.) terhadap *Streptococcus mutans.* *Makassar Dent. J.* 2018;7(1):26–29. 10.35856/mdj.v7i1.12

[24] Hamzah H Hertiani T Pratiwi SUT : The inhibition activity of tannin on the formation of mono-spesies and polymicrobial biofilm *Escherichia coli*, *Staphylococcus aureus, Pseudomonas aeruginosa,* and *Candida albicans.* *Trad. Med. J.* 2019;24(2):110–118. 10.35856/mdj.v7i1.12

[25] Mundada Y Gajare S Jankar A : Flexural strength of heat polymerized acrylic resin required with different reinforcing materials. *Int. J. Health Sci.* 2022;6(S7):2389–2396. 10.53730/ijhs.v6nS7.11867

[26] Abubakar AR Haque M : Preparation of medicinal plants: Basic extraction and fractionation procedures for experimental purposes. *J. Pharm. Bioallied Sci.* 2020;12(1):1–10. 10.4103/jpbs.JPBS_175_19 32801594 PMC7398001

[27] Morse DJ Wilson MJ Wei X : Modulation of *Candida albicans* virulence in in vitro biofilms by oral bacteria. *Lett. Appl. Microbiol.* 2019;68:337–343. 10.1111/lam.13145 30825340 PMC6849710

[28] Fujinami W Nishikawa K Ozawa S : Correlation between the relative abundance of oral bacteria and *Candida albicans* in denture and dental plaques. *J. Oral Biosci.* 2021;63:175–183. 10.1016/j.job.2021.02.003 33662564

[29] Majnooni MB Ghanadian SM Mojarrab M : Antibacterial, antibiofilm, antiswarming, and antioxidant activities of flavonoids isolated from *Allium colchicifolium* leaves. *J. Food Biochem.* 2023;2023:1–14. 10.1155/2023/5521661

[30] Nadeem SG Shafiq A Hakim ST : Effect of growth media, pH, and temperature on yeast to hyphal transition in *Candida albicans.* *Open J. Med. Microbiol.* 2013;03:185–192. 10.4236/ojmm.2013.33028

[31] Atriwali T Azeem K Husain MF : Mechanistic understanding of *Candida albicans* biofilm formation and approaches for its inhibition. *Front. Microbiol.* 2021;12:638609. 10.3389/fmicb.2021.638609 33995297 PMC8121174

[32] Wulansari S Mintarjo DF : Efek ekstrak etanol biji alpukat ( *Persea americana*) terhadap biofilm *Candida albicans.* *JKGT.* 2023;5(1):239–243. 10.25105/jkgt.v5i1.17178

[33] Hassan Y Chew SY Than LTL : *Candida glabrata*: Pathogenicity and resistance mechanisms for adaptation and survival. *J. Fungi.* 2021;7(667):1–18. 10.3390/jof7080667 34436206 PMC8398317

[34] Vale-Silva L Sanglard D : Tipping the balance both ways: Drug resistance and virulence in *Candida glabrata.* *FEMS Yeast Res.* 2015;15(fov025):1–8. 10.1093/femsyr/fov025 25979690

[35] Farahyar S Zaini F Kordbacheh P : Expression of efflux pumps and fatty acid activator one genes in azole resistant *Candida glabrata* isolated from immunoccompromised patients. *Acta Med. Iran.* 2016;54(7):459–464. 27424018

[36] Yu S Chang Y Chen Y : Deletion of *ADA2* increases antifungal drug susceptibility and virulence in *Candida glabrata.* *Antimicrob. Agents Chemother.* 2018;62(3):e01924–e01917. 10.1128/AAC.01924-17 29311082 PMC5826168

[37] Wolff A Rodloff AC Vielkind P : Antimicrobial susceptibility of clinical oral isolates of *Actinomyces* spp. *Microorganisms.* 2022;10(125):1–11. 10.3390/microorganisms10010125 35056574 PMC8779083

[38] Steininger C Willinger B : Resistance patterns in clinical isolates of pathogenic *Actinomyces* species. *J. Antimicrob. Chemother.* 2016;71:422–427. 10.1093/jac/dkv347 26538502

[39] Calosa BT Sugiaman VK Pranata N : Comparison of antibacterial activity of both seeds and leaves ethanol extract of avocado ( *Persea americana Mill.*) against *Streptococcus mutans.* *MDJ.* 2023;12(1):38–42. 10.35856/mdj.v12i1.629

[40] Liu S Sun Y Liu Y : Genomic and phenotypic characterization of *Streptococcus mutans* isolates suggets key gene clusters in regulating its interaction with *Streptococcus gordonii.* *Front. Microbiol.* 13:945108. 10.3389/fmicb.2022.945108 36033899 PMC9416482

[41] Campos J Pires MF Sousa M : Unveiling the relevance of the oral cavity as a *Staphylococcus aureus* colonization site and potential source of antimicrobial resistance. *Pathogens.* 2023;12(765):1–10. 10.3390/pathogens12060765 37375455 PMC10304336

[42] Montelongo-Jauregui D Lopez-Ribot JL : Candida interactions with the oral bacterial microbiota. *J. Fungi.* 2018;4(122):1–15. 10.3390/jof4040122 30400279 PMC6308928

[43] Santosa CM Rosyadi I Arifianto D : Kajian kliniko-patologik dan antimikroba ekstrak biji alpukat ( *Persea americana* Mill.). *Jurnal Sain Veteriner.* 2019;37(2):160–165. 10.22146/jsv.40445

[44] Morelli VG Oliveira VC Vasconcelos GLL : Effect of effervescent tablets on removable partial denture hygiene. *Am. J. Dent.* 2023;36(2):75–80. 37076296

[45] O’Brien TJ Figueroa W Welch M : Decreased efficacy of antimicrobial agents in polymicrobial environment. *ISME J.* 2022;16:1694–1704. 10.1038/s41396-022-01218-7 35304578 PMC9213441

[46] Kart D Tavernier S Acker H : Activity of disinfectants against multispecies biofilms formed by *Staphylococcus aureus*, *Candida albicans*, and *Psedomonas aeruginosa.* *Biofouling.* 2014;30(3):377–383. 10.1080/08927014.2013.878333 24579656

[47] Anju VT Busi S Imchen M : Polymicrobial infections and biofilms: Clinical significance and eradication strategies. *Antibiotics.* 2022;11(1731):1–31. 10.3390/antibiotics11121731 36551388 PMC9774821

[48] Olson ML Jayaraman A Kao KC : Relative abundances of *Candida albicans* and *glabrata* in in vitro coculture biofilms impact and formation. *Appl. Environ. Microbiol.* 2018;84(8):e02769–e02717. 10.1128/AEM.02769-17 29427422 PMC5881068

[49] Brunke S Hube B : Two unlike cousins: *Candida albicans* and *C. glabrata* infection strategies. *Cell. Microbiol.* 2013;15(5):701–708. 10.1111/cmi.12091 23253282 PMC3654559

[50] Bernard C Girardot M Imbert C : *Candida albicans* interaction with gram-positive bacteria within interkingdom biofilms. *J. Mycol. Médicale.* 2020;30(1):1–8. 10.1016/j.mycmed.2019.100909 31771904

[51] Diaz PI Strausbaugh LD Dongari-Bagtzoglou : Fungal-bacterial interactions and their relevance to oral health: linking the clinic and the bench. *Front. Cell. Infect. Microbiol.* 2014;4(101):1–6. 10.3389/fcimb.2014.00101 25120959 PMC4114182

[52] Hu Y Niu Y Ye X : *Staphylococcus aureus* synergized with *Candida albicans* to increase the pathogenesis and drug resistance in cutaneous abscess and peritonitis murine models. *Pathogens.* 2021;10(1036):1–17. 10.3390/pathogens10081036 34451500 PMC8398722

[53] Guo Y Wei C Liu C : Inhibitory effects of oral *Actinomyces* on the proliferation, virulence and biofilm formation of *Candida albicans.* *Arch. Oral Biol.* 2015;60:1368–1374. 10.1016/j.archoralbio.2015.06.015 26143096

[54] Uruén C Chopo-Ecuin G Tommassen J : Biofilms as promoters of bacterial antibiotic resistance and tolerance. *Antibiotics.* 2021;10(3):1–36. 10.3390/antibiotics10010003 33374551 PMC7822488

[55] Batoni G Maisetta G Esin S : Therapeutic potential of antimicrobial peptides in polymicrobial biofilm-associated infections. *Int. J. Mol. Sci.* 2021;22(482):1–24. 10.3390/ijms22020482 33418930 PMC7825036

[56] Vinha AF Moreira J Barreira SVP : Physicochemical parameters, phytochemical composition and antioxidant activity of algarvian avocado ( *Persea americana* Mill.). *J. Agric. Sci.* 2013;5(12):100–109. 10.5539/jas.v5n12p100

[57] Gutiérrez-Venegas G Gόmez-Mora JA Meraz-Rodríguez MA : Effect of flavonoids on antimicrobial activity of microorganisms present in dental plaque. *Heliyon.* 2019;5:e03013. 10.1016/j.heliyon.2019.e03013 31886429 PMC6921118

[58] Matilla-Cuenca L Gil C Cuesta S : Antibiofilm activity of flavonoids on staphylococcal biofilms through targeting BAP amyloids. *Nat. Res.* 2020;10(18968):18912–18968. 10.1038/s41598-020-75929-2 33144670 PMC7641273

[59] Dennis Nurliza C Savitri W : Antibacterial effect of ethanol extract of the avocado seed ( *Persea americana* Mill.) as an alternative root canal irrigants againts *Porphyromonas gingivalis* ( *in vitro*). *Int. J. App. Dent. Sci.* 2017;3(1):89–93. 2394-7497.

[60] Villanueva X Zhen L Ares JN : Effect of chemical modifications of tannins on their antimicrobial and antibiofilm effect against Gram-negative and Gram-positive bacteria. *Front. Microbiol.* 2023;13(987164):1–15. 10.3389/fmicb.2022.987164 36687646 PMC9853077

[61] Yan Y Li X Zhang C : Research progress on antibacterial activities and mechanisms of natural alkaloids: A review. *Antibiotics.* 2021;10(318):1–30. 10.3390/antibiotics10030318 33808601 PMC8003525

[62] Brahim MAS Fadli M Markouk M : Synergistic antimicrobial and antioxidant activity of saponins-rich extracts from *paronychia argentea* and *Spergularia marginata.* *European J. Med. Plants.* 2015;7(4):193–204. 10.9734/EJMP/2015/16597

[63] Monte J Abreu AC Borges A : Antimicrobial activity of selected phytochemicals against *Escherichia coli* and *Staphylococcus aureus* and their biofilms. *Pathogens.* 2014;3:473–498. 10.3390/pathogens3020473 25437810 PMC4243457

[64] Tamfu AN Ceylan O Cârâc G : Antibiofilm and anti-quorum sensing potential of cycloartane-type triterpene acids from Cameroonian grassland propolis: Phenolic profile and antioxidant activity of crude extract. *Molecules.* 2022;27(4872):1–19. 10.3390/molecules27154872 35956824 PMC9369644

[65] Kaypetch R Rudrakanjana P Tuangam P : Effects of two novel denture cleansers on mutispesies microbial biofilms, stain removal and the denture surface: An in vitro study. *BMC Oral Health.* 2023;23(852):1–12. 10.1186/s12903-023-03535-5 37951865 PMC10640750

[66] Lucena-Ferreira SC Cavalcanti IMG Cury AAB : Efficacy of denture cleansers in reducing microbial counts from removable partial dentures: A short-term clinical evaluation. *Braz. Dent. J.* 2013;24(4):353–356. 10.1590/0103-6440201302183 24173255

[67] Angela T : Avocado seed extract on inhibiting mono-species and polymicrobial biofilm.[Dataset]. *figshare.* 2024. 10.6084/m9.figshare.25996006

