# Supplementary material for: The effect of soaking heat-polymerized acrylic resin denture base in avocado seed extract ( Persea americana Mill.) on the inhibition of denture-plaque microorganisms biofilm growth
Source: F1000Res. 2025 Mar 25;13:933. Originally published 2024 Aug 20. [Version 3] doi: 10.12688/f1000research.152800.3 (PMC11806257; doi:10.12688/f1000research.152800.3)
Supplement: Peer review history

# Reviewer report: Reviewer response for version 3

**DOI:** 10.5256/f1000research.178613.r372991

**Published:** 2026-06-17

**Authors:** Veena Hegde

**Affiliations:** 1 Manipal Academy of Higher Education, Manipal, Karnataka, India

Do not have any comment.

Is the work clearly and accurately presented and does it cite the current literature?

Yes

If applicable, is the statistical analysis and its interpretation appropriate?

Yes

Are all the source data underlying the results available to ensure full reproducibility?

Yes

Is the study design appropriate and is the work technically sound?

Yes

Are the conclusions drawn adequately supported by the results?

Yes

Are sufficient details of methods and analysis provided to allow replication by others?

Yes

Reviewer Expertise:

Dental materials related to prosthodontics

I confirm that I have read this submission and believe that I have an appropriate level of expertise to confirm that it is of an acceptable scientific standard.

# Reviewer report: Reviewer response for version 2

**DOI:** 10.5256/f1000research.173600.r361435

**Published:** 2025-02-14

**Authors:** Veena Hegde

**Affiliations:** 1 Manipal Academy of Higher Education, Manipal, Karnataka, India

The effect of immersing Heat polymerized acrylic resin denture base in different concentration of avocado seeds extract is done stystematically to determine the minimum biofilm inhibitory concentration.

 Difference in minimum biofilm inhibitory concentration of avocado extract against polymicrobial biofilm and  monospecies biofilm is well justified.

 Overall, this is a clear, concise, and well-written manuscript.

 Strengths of the Study:

1. Innovative Use of Natural Extracts:
  - The study explores the antimicrobial properties of avocado seed extract, a novel approach within the field of denture care. This investigation aligns with increasing interest in natural products as alternatives to conventional antimicrobial agents, potentially benefiting oral health practices .
2. Comprehensive Testing Across Multiple Concentrations:
  - It systematically examines a variety of concentrations (5%, 10%, 15%, and 20%), providing a clear understanding of the dose-response relationship regarding biofilm inhibition. This thorough assessment is vital for identifying the most effective concentration for practical applications ..
3. Significant Results with Clinical Relevance:
  - The study's findings indicate that a 20% concentration of avocado seed extract can inhibit more than 50% of polymicrobial biofilm growth, suggesting a promising natural alternative for denture hygiene. This has important implications for improving oral health among denture wearers .
4. Implications for Future Research:
  - The research offers a foundation for further studies into the phytochemical constituents of avocado seed extract, which could lead to the identification of specific antimicrobial compounds. This paves the way for more targeted and effective natural products in dental care.
  -  Weaknesses of the Study:
5. Extraction Method Concerns:
  - The use of 70% ethanol as a solvent in the extraction process may confound the results, as the observed antimicrobial effects might result from the ethanol rather than the avocado seed compounds alone. This raises questions about the specificity of the extract's efficacy .
6. Limited Representation of Real-World Conditions:
  - Conducting the study in vitro means the findings may not fully translate to the complexities of the oral cavity in actual denture wearers, where additional factors (such as saliva, diet, and oral microbiome dynamics) play significant roles in biofilm formation .
  - Understanding the spectrum of microbial inhibition would provide a better assessment of the extract’s antibacterial profile .

Summary

 The study presents a promising exploration of avocado seed extract's antimicrobial properties for denture care, showcasing innovative methods and significant findings that could enhance oral health. While there are notable strengths, including its systematic approach and robust statistical analysis, addressing the extraction method, real-world applicability, and microbial profiling would enhance the study's overall impact and relevance. Balancing these strengths and weaknesses will contribute to informed discussions regarding the potential use of natural remedies in dental hygiene practices.

Is the work clearly and accurately presented and does it cite the current literature?

Yes

If applicable, is the statistical analysis and its interpretation appropriate?

Yes

Are all the source data underlying the results available to ensure full reproducibility?

Yes

Is the study design appropriate and is the work technically sound?

Yes

Are the conclusions drawn adequately supported by the results?

Yes

Are sufficient details of methods and analysis provided to allow replication by others?

Yes

Reviewer Expertise:

Dental materials related to prosthodontics

I confirm that I have read this submission and believe that I have an appropriate level of expertise to confirm that it is of an acceptable scientific standard.

# Response

**Published:** 2025-02-23

**Authors:** Thalia Angela

**Affiliations:** Department of Dentistry, University of Sumatera Utara, Medan, North Sumatra, Indonesia

We would like to express our gratitude for the reviewer’s approval of our research and for the mentioned strengths and weaknesses of our study.

# Reviewer report: Reviewer response for version 2

**DOI:** 10.5256/f1000research.173600.r359394

**Published:** 2025-02-07

**Authors:** Norlela Yacob

**Affiliations:** 1 Universiti Sains Islam Malaysia, Nilai, Malaysia

Abstract does not contain objective of study. please add.

 Result in abstract is confusing:  There was a significant effect of soaking HPA resin in avocado seed extract on the inhibition of mono-species and polymicrobial biofilms with a value of p<0.001 (p<0.05).- which one is correct?

 Introduction was good.

 Methodology was satisfactory.

 Result are satisfactory. 

 Table 3. asteriks mean what? need to explain further

 Result in Table 6. should have post hoc test that shows which concentration does have significant different. 

 Figure 2. The image does not self-explanatory. please improve the label and what the findings.

 discussion was good. 

 Where is the conclusion? it would be good to recommend the clinical application of this avocado solution as denture cleanser.

Is the work clearly and accurately presented and does it cite the current literature?

Yes

If applicable, is the statistical analysis and its interpretation appropriate?

Partly

Are all the source data underlying the results available to ensure full reproducibility?

Yes

Is the study design appropriate and is the work technically sound?

Yes

Are the conclusions drawn adequately supported by the results?

Partly

Are sufficient details of methods and analysis provided to allow replication by others?

Yes

Reviewer Expertise:

micorbial adherence, denture microbiome, digtial denture, 3D printing

I confirm that I have read this submission and believe that I have an appropriate level of expertise to confirm that it is of an acceptable scientific standard, however I have significant reservations, as outlined above.

# Response

**Published:** 2025-02-23

**Authors:** Thalia Angela

**Affiliations:** Department of Dentistry, University of Sumatera Utara, Medan, North Sumatra, Indonesia

First and foremost, we would like to thank the reviewers for the reviews given to improve the content and writing of the articles. We have made the changes accordingly in the new version and we would also like to humbly respond to some of the reviews given.

 Abstract does not contain objective of study. please add.

**Response: **We have added the objective of the study on the last sentence of the abstract background.

 Result in abstract is confusing:  There was a significant effect of soaking HPA resin in avocado seed extract on the inhibition of mono-species and polymicrobial biofilms with a value of p<0.001 (p<0.05) - which one is correct?

**Response: **Both are correct. The result from our ANOVA test specifically showed a value of p<0.001. The value in brackets were for further emphasis that the p value is below 0.05, which 0.05 is the most commonly set standard of significant levels. A p-value below 0.05 means there was a significant effect of soaking HPA resin in avocado seed extract on the inhibition of mono-species and polymicrobial biofilms

 Introduction was good. Methodology was satisfactory. Result are satisfactory. Discussion was good.

**Response: **We would like to express our gratitude for the reviewer’s approval of the sections mentioned.

 Table 3. asteriks mean what? need to explain further

**Response: **We have added the meaning of the asterisk right below the table

 Result in Table 6. should have post hoc test that shows which concentration does have significant different

**Response: **We have done the post hoc test, however we could not insert the whole data in the journal as they were too many data to be included. Despite that, we have added a brief explanation of the post hoc test results in the result section, and we have inserted the full complete data which can be accessed from the link in the data availability section.

 Figure 2. The image does not self-explanatory. please improve the label and what the findings.

**Response:** We have further explained the image (Figure 2)

 Where is the conclusion? it would be good to recommend the clinical application of this avocado solution as denture cleanser.

**Response: **We have added the conclusion Section.

# Reviewer report: Reviewer response for version 2

**DOI:** 10.5256/f1000research.173600.r336000

**Published:** 2024-11-21

**Authors:** Nada Z. Mohammed, Suhad M. Hamdoon

**Affiliations:** 1 Department of Prosthodontics, College of Dentistry, University of Mosul (Ringgold ID: 108489), Mosul, Nineveh Governorate, Iraq; 2 Department of Basic Dental Sciences, College of Dentistry, University of Mosul (Ringgold ID: 108489), Mosul, Nineveh, Iraq

The authors do the requested corrections.

Is the work clearly and accurately presented and does it cite the current literature?

Yes

If applicable, is the statistical analysis and its interpretation appropriate?

Partly

Are all the source data underlying the results available to ensure full reproducibility?

Partly

Is the study design appropriate and is the work technically sound?

Partly

Are the conclusions drawn adequately supported by the results?

Yes

Are sufficient details of methods and analysis provided to allow replication by others?

Yes

Reviewer Expertise:

Prosthetic dentistry, dental materials, Microbiology

We confirm that we have read this submission and believe that we have an appropriate level of expertise to confirm that it is of an acceptable scientific standard.

# Response

**Published:** 2025-02-23

**Authors:** Thalia Angela

**Affiliations:** Department of Dentistry, University of Sumatera Utara, Medan, North Sumatra, Indonesia

We would like to express our gratitude for the reviewer’s approval of our research and for the previous review that has been given which has helped us improve our research paper.

# Reviewer report: Reviewer response for version 1

**DOI:** 10.5256/f1000research.167602.r320505

**Published:** 2024-10-29

**Authors:** Prabha S Newaskar

**Affiliations:** 1 Department of Prosthodontics, Pravara Institute of Medical Sciences, Ahmednagar, Maharashtra, India

The current in-vitro study is based on denture plaque microorganism inhibition by avocado seed extract on heat-cured denture base resin. The author considered the 5%, 10%, 15% & 20% concentrations of the same extract and two groups of controls. for the study. and concluded that a 20% concentration was more effective than other concentrations. The following are suggestions for improvement of the current manuscript:

 1. Abstract: Ensure that the abstract concisely summarizes the key findings. The methodology, key results, and conclusion can be streamlined further to highlight the core contributions without too much technical detail.

 2. Methodology: Include any steps taken to mitigate bias or ensure reproducibility. For instance, if there were any controls (such as positive and negative controls), clearly define how they were handled to ensure the integrity of the experiment. Specify more details about the process of ensuring the sterility of HPA resin disks or provide a brief explanation of the significance of the preparation technique.

 3. Result: Add more visual aids (tables, charts, or graphs) to clarify results. This will make it easier for readers to interpret the data. provide context to the statistical findings. Instead of just reporting p-values, discuss the biological or practical significance of these results.

 4. Discussion: Ensure that each point is directly tied back to the research question. Avoid over-explaining background information already provided in the introduction. Provide a more detailed comparison between your findings and previous studies. Highlight areas where your results diverge or align with other research to emphasize the novelty or contribution of your work.

 5. Conclusion: You may consider discussing the potential limitations of your study (e.g., the in vitro setting) and suggest avenues for future research.

 6. Reference & citations: Double-check all citations to ensure they adhere to the journal's guidelines (e.g., proper format, all sources accounted for). Ensure recent and relevant literature is cited, particularly on the antimicrobial activity of avocado seed extracts.

 7. Ethical consideration: If the study involves the handling of microorganisms, briefly outline the safety measures taken to ensure proper disposal and handling, in compliance with biosafety standards.

 8. Language & grammar: Revise for grammatical consistency. For instance, avoid shifting tenses within the same section. Avoid redundancy. For example, once the MBIC50 is defined, you do not need to explain it repeatedly in different sections.

Is the work clearly and accurately presented and does it cite the current literature?

Partly

If applicable, is the statistical analysis and its interpretation appropriate?

Yes

Are all the source data underlying the results available to ensure full reproducibility?

Partly

Is the study design appropriate and is the work technically sound?

Yes

Are the conclusions drawn adequately supported by the results?

Partly

Are sufficient details of methods and analysis provided to allow replication by others?

Partly

Reviewer Expertise:

Prosthodontics, Dental materials, Implants, Maxillofacial prosthodontics, Systematic reviews, Fixed prosthodontics, Removable prosthodontics.

I confirm that I have read this submission and believe that I have an appropriate level of expertise to confirm that it is of an acceptable scientific standard, however I have significant reservations, as outlined above.

# Response

**Published:** 2025-02-23

**Authors:** Thalia Angela

**Affiliations:** Department of Dentistry, University of Sumatera Utara, Medan, North Sumatra, Indonesia

**First and foremost, we would like to thank the reviewers for the reviews given to improve the content and writing of the articles. We would like to apologize for the long update of the article new version and we are very grateful for your patience. We have made the changes accordingly in the new version and we would also like to humbly respond to some of the reviews given.**

 Abstract: Ensure that the abstract concisely summarizes the key findings. The methodology, key results, and conclusion can be streamlined further to highlight the core contributions without too much technical detail.

**R: We have tried to streamline the conclusion section of the abstract. However, for the methodology and results, we couldn’t do much especially with the previous version review that suggest us to add the name of the test and the statistical tests done on this study. We hope the small changes in the abstract could be sufficient in this revised manuscript.**

 Methodology: Include any steps taken to mitigate bias or ensure reproducibility. For instance, if there were any controls (such as positive and negative controls), clearly define how they were handled to ensure the integrity of the experiment. Specify more details about the process of ensuring the sterility of HPA resin disks or provide a brief explanation of the significance of the preparation technique.

**R: We have added additional explanation on the process of HPA resin disks sterility and the handling of the controls.**

 Result: Add more visual aids (tables, charts, or graphs) to clarify results. This will make it easier for readers to interpret the data. provide context to the statistical findings. Instead of just reporting p-values, discuss the biological or practical significance of these results.

**R: We couldn’t add more visual aids as we have already included the tables in the result section, in which each table is marked with asterisk to signify certain attention to data. We have also added more data interpretation according to our statistical findings.**

 Discussion: Ensure that each point is directly tied back to the research question. Avoid over-explaining background information already provided in the introduction. Provide a more detailed comparison between your findings and previous studies. Highlight areas where your results diverge or align with other research to emphasize the novelty or contribution of your work.

**R: We have ensured our point of discussion has referred back to the research purpose and deleted any over-explaining background information.**

 Conclusion: You may consider discussing the potential limitations of your study (e.g., the in vitro setting) and suggest avenues for future research.

**R: We have added conclusion section and a separate section for the study limitation in the revised manuscript.**

 Reference & citations: Double-check all citations to ensure they adhere to the journal's guidelines (e.g., proper format, all sources accounted for). Ensure recent and relevant literature is cited, particularly on the antimicrobial activity of avocado seed extracts.

**R: We have double-checked our reference and citations.**

 Ethical consideration: If the study involves the handling of microorganisms, briefly outline the safety measures taken to ensure proper disposal and handling, in compliance with biosafety standards.

**R: We have added the brief outline of microorganism proper disposal and handling.**

 Language & grammar: Revise for grammatical consistency. For instance, avoid shifting tenses within the same section. Avoid redundancy. For example, once the MBIC50 is defined, you do not need to explain it repeatedly in different sections.

**R: We have revised our grammatical errors and avoided redundancy.**

# Reviewer report: Reviewer response for version 1

**DOI:** 10.5256/f1000research.167602.r320506

**Published:** 2024-10-04

**Authors:** Mervat E. Abd-Ellah

**Affiliations:** 1 Department of Prosthodontics, Faculty of Dentistry, Alexandria University, Alexandria, Alexandria Governorate, Egypt; 2 Prothodontics, Alexandria University Faculty of Dentistry, Alexandria, Alexandria Governorate, Egypt

The work being reviewed is entitled “ The effect of soaking heat-polymerized acrylic resin denture base in avocado seed extract on the inhibition of denture-plaque microorganisms biofilm growth”  

 This study aimed to evaluate the effect of avocado seeds on denture-plaque microorganism biofilm on Heat polymerized acrylic (HPA) resins. It was concluded that 20% avocado seed extract was effective in inhibiting polymicrobial biofilm because it was able to inhibit more than 50% polymicrobial biofilm. 

 There are certain limitations for approving this study;

 - First, it was mentioned that “The avocado seed powder was put into a vessel and poured with 70% ethanol solvent” then may be the antimicrobial effect from the ethanol not the avocado seed powder and that makes the idea of the study not sound.

 - Second, the work partly cite the current literature as it does not justify the use of the used concentrations of  avocado seed extract clearly. It was mentioned that “Anggraini et al. (2017 [Ref - 1]) studied the inhibition zone of avocado seed extract at concentrations of 10%, 20%, 40%, 80%, 100% on the growth of *C. albicans*, and found that the 10% concentration was the most effective concentration in inhibiting *C. albicans.* ^22^ Another study by Talib et al. (2018 [Ref - 2]) tested the effectiveness of avocado seed extract in inhibiting *Streptococcus mutans* at concentrations of 2%, 4%, 6%, 8%, 10% and found that the most effective concentration was 10%. ^23^” It was already stated that 10% is the most effective concentration, then it was useless to test the same concentration that was used before. This is an unnecessary repetition. Moreover, it was concluded in the current study that 20% avocado seed extract was effective in inhibiting polymicrobial biofilm but it was not justified way the difference in percentage of avocado seed extract from the literature that stated that 10 % was the most effective concentration in inhibiting *C. albicans*

*- *Third, it was mentioned that “Sterile HPA resin discs were preconditioned for 24 hours by immersion in artificial saliva.” You did not say how the process of sterilization happened. Please clarify.

Is the work clearly and accurately presented and does it cite the current literature?

Partly

If applicable, is the statistical analysis and its interpretation appropriate?

I cannot comment. A qualified statistician is required.

Are all the source data underlying the results available to ensure full reproducibility?

No source data required

Is the study design appropriate and is the work technically sound?

No

Are the conclusions drawn adequately supported by the results?

Partly

Are sufficient details of methods and analysis provided to allow replication by others?

Yes

Reviewer Expertise:

prosthodontics

I confirm that I have read this submission and believe that I have an appropriate level of expertise to state that I do not consider it to be of an acceptable scientific standard, for reasons outlined above.

## References

[1] : EFEKTIVITAS KOMBINASI EKSTRAK DAUN SIRIH MERAH (Piper Crocatum) DAN EKSTRAK BIJI ALPUKAT (Persea americana) DALAM MENGHAMBAT PERTUMBUHAN Candida albicans. *Jurnal Kimia Riset* .2017;2(2) : 10.20473/jkr.v2i2.6196 10.20473/jkr.v2i2.6196

[2] : Efektivitas antibakteri ekstrak biji alpukat (Persea americana Mill.) terhadap Streptococcus mutans (Antibacterial effectiveness of avocado seed (Persea americana Mill.) extract on Streptococcus mutans). *Makassar Dental Journal* .2018;7(1) : 10.35856/mdj.v7i1.12 10.35856/mdj.v7i1.12

# Response

**Published:** 2024-10-18

**Authors:** Thalia Angela

**Affiliations:** Department of Dentistry, University of Sumatera Utara, Medan, North Sumatra, Indonesia

**First and foremost, we would like to thank the reviewers for the reviews given to improve the content and writing of the articles. We have made the changes accordingly in the new version and we would also like to humbly respond to some of the reviews given.**

**Reviewer comments and Angela et al. responses (R.)**

 First, it was mentioned that “The avocado seed powder was put into a vessel and poured with 70% ethanol solvent” then may be the antimicrobial effect from the ethanol not the avocado seed powder and that makes the idea of the study not sound.

**R: We would like to explain the use of 70% ethanol solvent when it was poured inside the vessel containing the avocado seed powder. According to Abubakar et al. (2020), solvent is also known as menstruum which is defined as a liquid chosen for an effective extraction process. ^1^ The solvent used in this study is ethanol which is a polar solvent that is used to extract polar compounds such as flavonoids and polyphenols. The reason we are using 70% ethanol (water-ethanol) instead of water only is that according to Plaskova et al. (2023), all the bioactive molecules from the plant material cannot be extracted using only single solvent, instead binary solvent mixture such as water and organic solvent are recommended. The study claimed that water-ethanol solvent shows a much better extraction of polyphenols than any single solvent. ^2^ Another study that supported this claim is a study by Munthe et al. (2023) which concluded that 70% ethanolic extract of avocado seeds using maceration technique showed a strong antioxidant activity. ^3^**

**In our study, at the end of extraction process, the filtered liquid avocado seed extract underwent evaporation on a rotary evaporator which removed the solvents used for extracting the avocado seeds, leaving behind a very thick extract. Plaskova et al. (2023) did mentioned that some residual solvents might remain unintentionally, however the level of the remaining solvent is limited. The study has also presented a table showcasing the maximum limitation of the solvent which can be further read in the published paper. ^2^ The thick extract that our study has gained are then diluted using aquadest, hence the negative control of our study is also aquadest because it can be said that through the evaporation process, the ethanol solvent has little to no effect in contributing to the antimicrobial effect.**

**References**

1. **Abubakar AR, Haque M. Preparation of medicinal plants: basic extraction and fractionation procedures for experimental purposes. *J Pharm Bioallied Sci*. 2020;12(1):1-10. doi: 10.4103/jpbs.JPBS_175_19**
2. **Plaskova A, Mlcek. New insights of the application of water or ethanol-water plant extract rich in active compounds in food. *Front Nutr*. 2023;10:1118761. doi: 10.3389/fnut.2023.1118761**
3. **Munthe SWN, Riskianto R, Juvi D, Novia J. Antioxidant, total phenolic, and total flavonoid of 70% ethanol extract of avocado seeds ( *Persea americana *Mill.). *Pharmacogn J. *2023;15(4):599-605. doi: 10.5530/pj.2023.15.126**

Second, the work partly cite the current literature as it does not justify the use of the used concentrations of avocado seed extract clearly. It was mentioned that “Anggraini et al. (2017 [Ref - 1]) studied the inhibition zone of avocado seed extract at concentrations of 10%, 20%, 40%, 80%, 100% on the growth of C. albicans, and found that the 10% concentration was the most effective concentration in inhibiting C. albicans. ^22^ Another study by Talib et al. (2018 [Ref - 2]) tested the effectiveness of avocado seed extract in inhibiting Streptococcus mutans at concentrations of 2%, 4%, 6%, 8%, 10% and found that the most effective concentration was 10%. ^23^” It was already stated that 10% is the most effective concentration, then it was useless to test the same concentration that was used before. This is an unnecessary repetition. Moreover, it was concluded in the current study that 20% avocado seed extract was effective in inhibiting polymicrobial biofilm but it was not justified way the difference in percentage of avocado seed extract from the literature that stated that 10% was the most effective concentration in inhibiting C. albicans.

**R: We would like to explain the reason why we included those previous studies in our Introduction section and why we did our research based on the concentrations that the previous studies have concluded. Denture plaque that is formed in the denture wearers appliances is not a mono-species biofilm but a polymicrobial one.  O’Donnell et al. (2015) declared that denture plaque consists of *Candida *spp. which co-aggregates with bacteria. ^1^ The previous studies we included in our paper are only done on planktonic bacteria and fungal, not polymicrobial biofilm. Meanwhile a study by Hamzah et al. (2019) has found that a much higher concentration is needed to inhibit polymicrobial biofilm than mono-species biofilms. Not to mention, the previous studies we included our papers are done on *C. albicans *and *S. mutans *while our paper actually studies the effect of avocado seed on *C. albicans, C. glabrata, A. odontolyticus, S. gordonii, S. aureus*, both mono-species and polymicrobial biofilms *. *Hence, our study still used the previous established effective concentration (10%) with varieties that centered on the concentration from previous studies (5%, 15%, 20%). ^2^ We also still studied the effect of avocado seed on the mono-species biofilms to not only know which concentration can be determined as the MBIC _50_ on each mono-species biofilms, but also to see whether there is truly a difference between the MBIC _50 _of each mono-species biofilm and the polymicrobial biofilm. The result of our study, which summary can be seen on our Abstract section, has shown that MBIC _50 _for each mono-species are different from the previous studies’ concentration. This shows that our study did not do any unnecessary repetition.**

**On a side note, we have added further explanation on the research gap in our introduction section. We hope it could add more clearance on the matter. We truly appreciate the feedback given by the reviewer.**

**References**

1. **O’Donnell LE, Robertson D, Nile CJ, et al.: The oral microbiome of denture wearers is influenced by levels of natural dentition. PLoS One. 2015;10(9):e0137717. 26368937 10.1371/journal.pone.0137717 PMC4569385**
2. **Hamzah H, Hertiani T, Pratiwi SUT, et al.: The inhibition activity of tannin on the formation of mono-spesies and polymicrobial biofilm Escherichia coli, Staphylococcus aureus, Pseudomonas aeruginosa, and Candida albicans. Trad. Med. J. 2019;24(2):110–118. 10.35856/mdj.v7i1.12**

Third, it was mentioned that “Sterile HPA resin discs were preconditioned for 24 hours by immersion in artificial saliva.” You did not say how the process of sterilization happened. Please clarify.

**R: We have clarified the sterilization process of the HPA resin discs in the Methods section.**

# Reviewer report: Reviewer response for version 1

**DOI:** 10.5256/f1000research.167602.r320510

**Published:** 2024-10-03

**Authors:** Nada Z. Mohammed, Suhad M. Hamdoon

**Affiliations:** 1 Department of Prosthodontics, College of Dentistry, University of Mosul (Ringgold ID: 108489), Mosul, Nineveh Governorate, Iraq; 2 Department of Basic Dental Sciences, College of Dentistry, University of Mosul (Ringgold ID: 108489), Mosul, Nineveh, Iraq

Abstract

 - The Methods are concise, it needs to be more informative, add the name of tests used.

 -You should add a concise information about the statistical analysis that used in the study.

 Introduction

 -The introduction is informative but lacks a clear statement of the research gap and aim of the research.

 Methods

 -The methodology section is detailed but could benefit from sequential numbering of the subheadings for better organization

 - There are many paragraphs without reference ; you should add the reference.

 - In Avocado seed extraction procedure ; Chromatography also should be carried out to identified the extract composition.

 - Mention the name of  medium used to culture *Actinomyces odontolyticus* on fastidious anaerobe agar.

 - In Biofilm formation on heat polymerized acrylic resin disc ; How much the amount of polymicrobial biofilm was added.

 - Inhibition Value%=[(ControlOD_SampleOD)÷-ControlOD]×100; clarify if the subtracted control was the negative or positive ?

 -Statistical analysis required more clarification  as mentioned by adding table for the inhibition value for each extract treatment group obtained by the inhibition formula to be compared directly  

 Result

 -The results are presented clearly; but it could be improved; the results display need to be more consistent  without excessively repeating the data. Display the main finding and explain the reason leads to this result

 -Please insert table contain the inhibition value obtained by the inhibition formula

 Discussion

 -The authors should enrich the discussion by the analysis and interpreting the obtained results and by comparing the obtained results with what reported in other papers.

 -The limitations of the studies should be presented.

 -The clinical relevance should be further highlighted.

 Tables

 -The MBIC50 of avocado seed extract and its effect on polymicrobial biofilm; the 50.81_8.32%. value must be placed in table.

 References

 There are too many references. Remove the outdated and unnecessary references .

Is the work clearly and accurately presented and does it cite the current literature?

Yes

If applicable, is the statistical analysis and its interpretation appropriate?

Partly

Are all the source data underlying the results available to ensure full reproducibility?

Partly

Is the study design appropriate and is the work technically sound?

Partly

Are the conclusions drawn adequately supported by the results?

Yes

Are sufficient details of methods and analysis provided to allow replication by others?

Yes

Reviewer Expertise:

Prosthetic dentistry, dental materials, Microbiology

We confirm that we have read this submission and believe that we have an appropriate level of expertise to confirm that it is of an acceptable scientific standard, however we have significant reservations, as outlined above.

# Response

**Published:** 2024-10-18

**Authors:** Thalia Angela

**Affiliations:** Department of Dentistry, University of Sumatera Utara, Medan, North Sumatra, Indonesia

First and foremost, we would like to thank the reviewers for providing necessary reviews to improve the content and writing of the articles. We have made changes in the new version of the article according to the reviews given.

 Reviewer comments and Angela et al. responses (R.)

 Abstract

 1. The Methods are concise, it needs to be more informative, add the name of tests used.

 2. You should add a concise information about the statistical analysis that used in the study

 R: We have included the name of the test (microtiter biofilm plate assay) and the statistical analysis used in the study as concise as possible.

 Introduction

 The introduction is informative but lacks a clear statement of the research gap and aim of the research.

 R: We have given a much clearer statements on the research gap and the research aim which can be seen on the last paragraph of the introduction section.

  Methods

 1. The methodology section is detailed but could benefit from sequential numbering of the subheadings for better organization

 R: We have added sequential numbering on the methods section.

 2. There are many paragraphs without reference; you should add the reference.

 R: We have added necessary references on the methods section.

 3. In Avocado seed extraction procedure; Chromatography also should be carried out to identified the extract composition.

 R: We do agree that the extract can use Chromatography to further identify the extract composition. In our research location, the only available Chromatography is Gas Chromatography Mass Spectrometry (GC-MS). However, we decided not to do the test during our research period as we only wanted to know the presence of flavonoids, polyphenols, alkaloids, and saponin in our avocado seed extract as many research have shown their antimicrobial properties in order to make sure that we fulfilled the purpose of our study which is to know the effect of avocado seed extract on denture plaque microorganisms grown on HPA resin. The two tests we have done, phytochemical examination and its quantitative tests, have shown the existence of those compound and specified their amount in the extract. Hence, we directly continue our research without doing Chromatography.

 4. Mention the name of medium used to culture *Actinomyces odontolyticus* on fastidious anaerobe agar.

 R: We have previously written the name of the medium used to culture *A. odontolyticus  *which is fastidious anaerobe agar which we supplemented with 5% (v/v) defibrinated bovine blood.

 5. In Biofilm formation on heat polymerized acrylic resin disc; How much the amount of polymicrobial biofilm was added.

 R: We have specified the amount of polymicrobial biofilm added which can be seen on the second subheading “Biofilm formation on heat polymerized acrylic resin disc”

 6. Inhibition Value%=[(ControlOD_SampleOD)÷-ControlOD]×100; clarify if the subtracted control was the negative or positive?

 R: We confirmed that the ControlOD for the inhibition value formula is positive, not negative.

 7. Statistical analysis required more clarification as mentioned by adding table for the inhibition value for each extract treatment group obtained by the inhibition formula to be compared directly

 R:  We have added the inhibition value table on the Result section.

 Result

 1. The results are presented clearly; but it could be improved; the results display need to be more consistent without excessively repeating the data. Display the main finding and explain the reason leads to this result

 R: We have removed the repetitive sentences.

 2. Please insert table contain the inhibition value obtained by the inhibition formula

 R: We have replaced the figures of inhibition value into tables (Table 3 dan Table 5).

 Discussion

 1. The authors should enrich the discussion by the analysis and interpreting the obtained results and by comparing the obtained results with what reported in other papers.

 R: We would like to apologize for our paper shortcoming. We have previously included as much other paper results as possible, however there is still limited data that can be used as comparison, especially for the microorganisms that we studied in this paper, such as *C. glabrata, A. odontolyticus, S. gordonii*.

 2. The limitations of the studies should be presented.

 R: We have presented the limitations of the study and made a separate section for it.

 3. The clinical relevance should be further highlighted.

 R: We have highlighted the clinical relevance which is written on the last sentence in the discussion section, above the study limitation.

 Tables

 The MBIC50 of avocado seed extract and its effect on polymicrobial biofilm; the 50.81_8.32%. value must be placed in table.

 R: We have added it to Table 5 in the results section.

 References

 There are too many references. Remove the outdated and unnecessary references

 R: We have removed the outdated and unnecessary references.
